# Supplementary figures and images for: Minimal twister sister-like self-cleaving ribozymes in the human genome revealed by deep mutational scanning
Source: eLife. 2024 Dec 5;12:RP90254. doi: 10.7554/eLife.90254 (PMC11620745; doi:10.7554/eLife.90254)

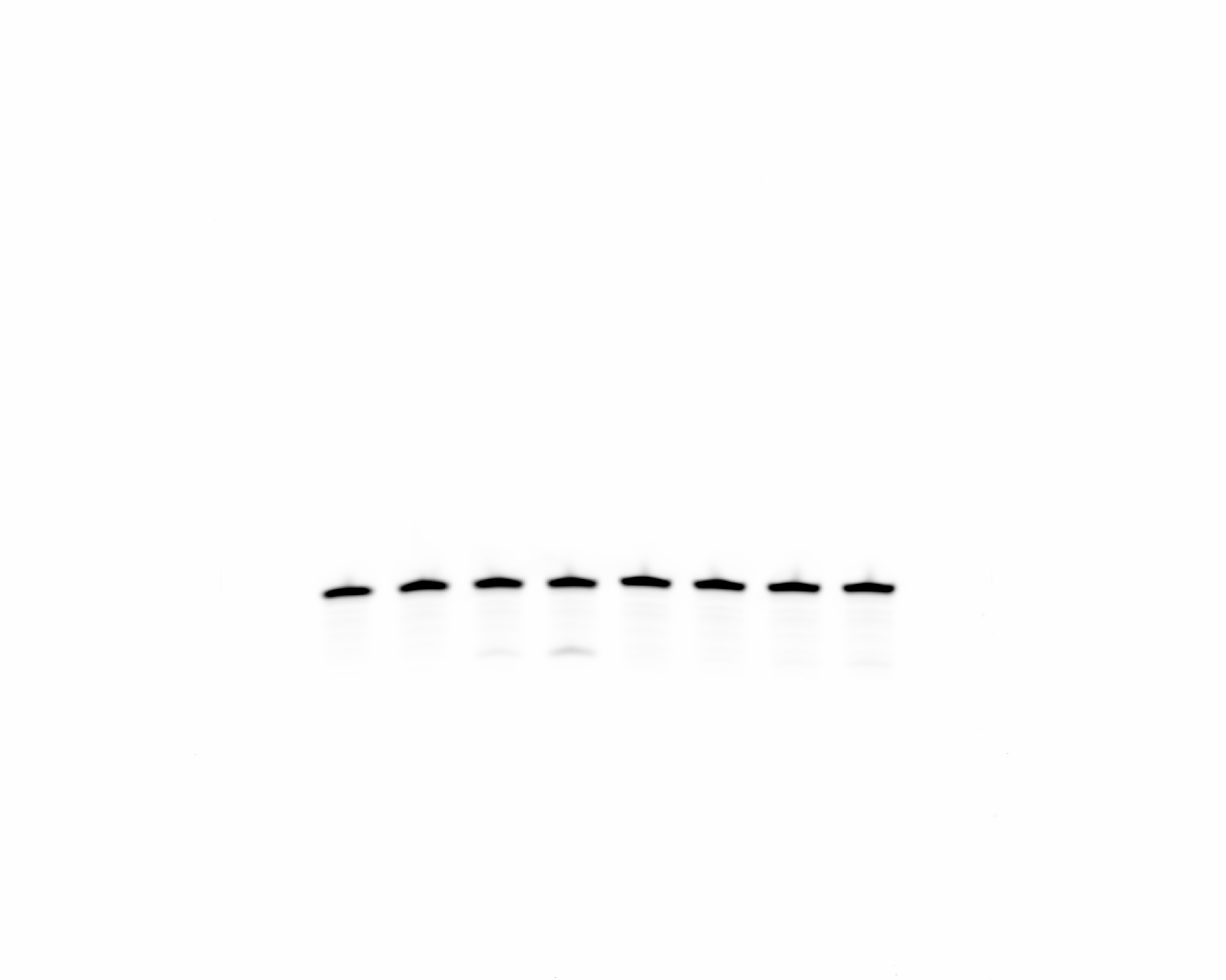

Supplement: Figure 4—source data 1. [file elife-90254-fig4-data1.zip › Figure 4C raw 2.tif]

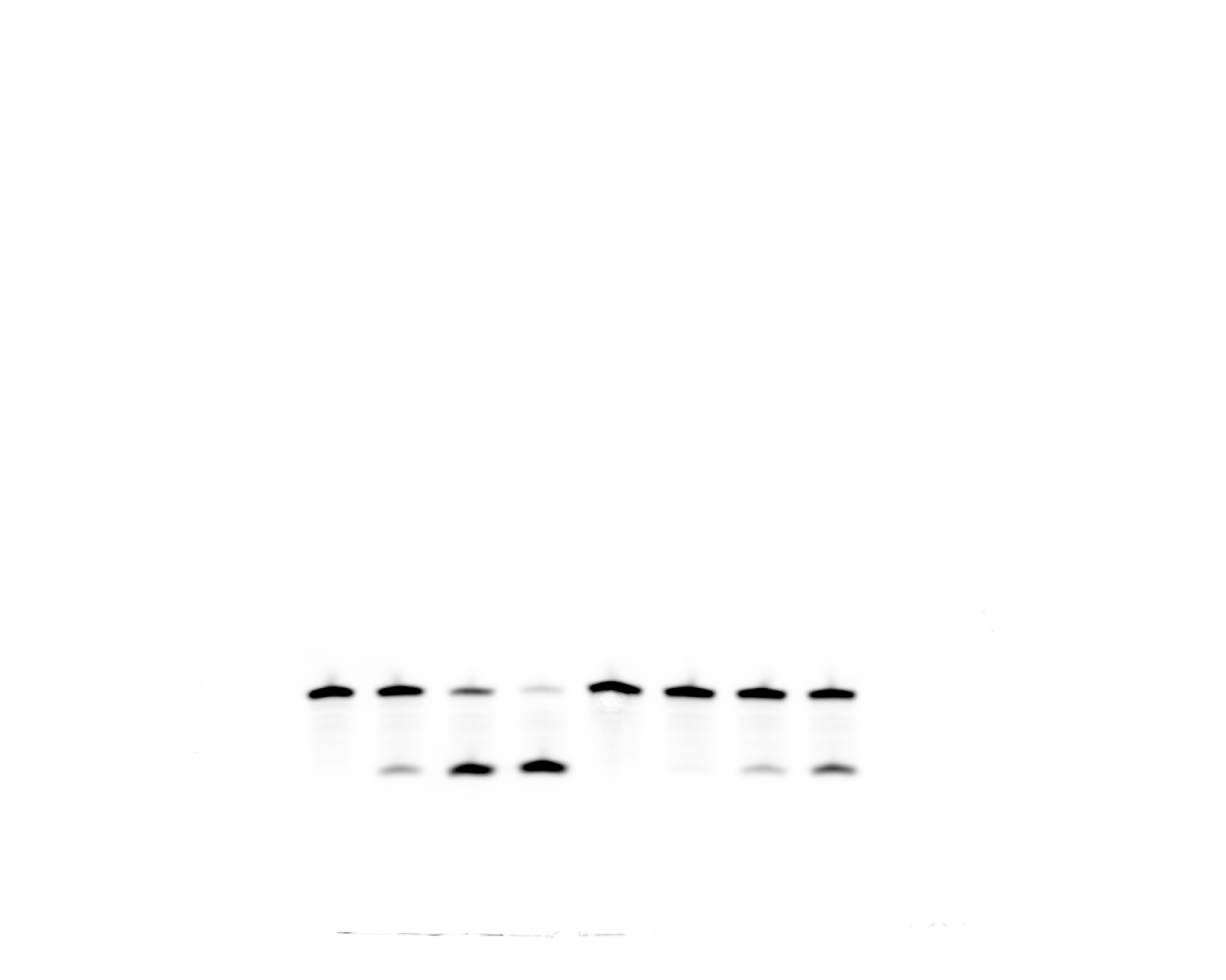

Supplement: Figure 4—source data 1. [file elife-90254-fig4-data1.zip › Figure 4C raw 3.tif]

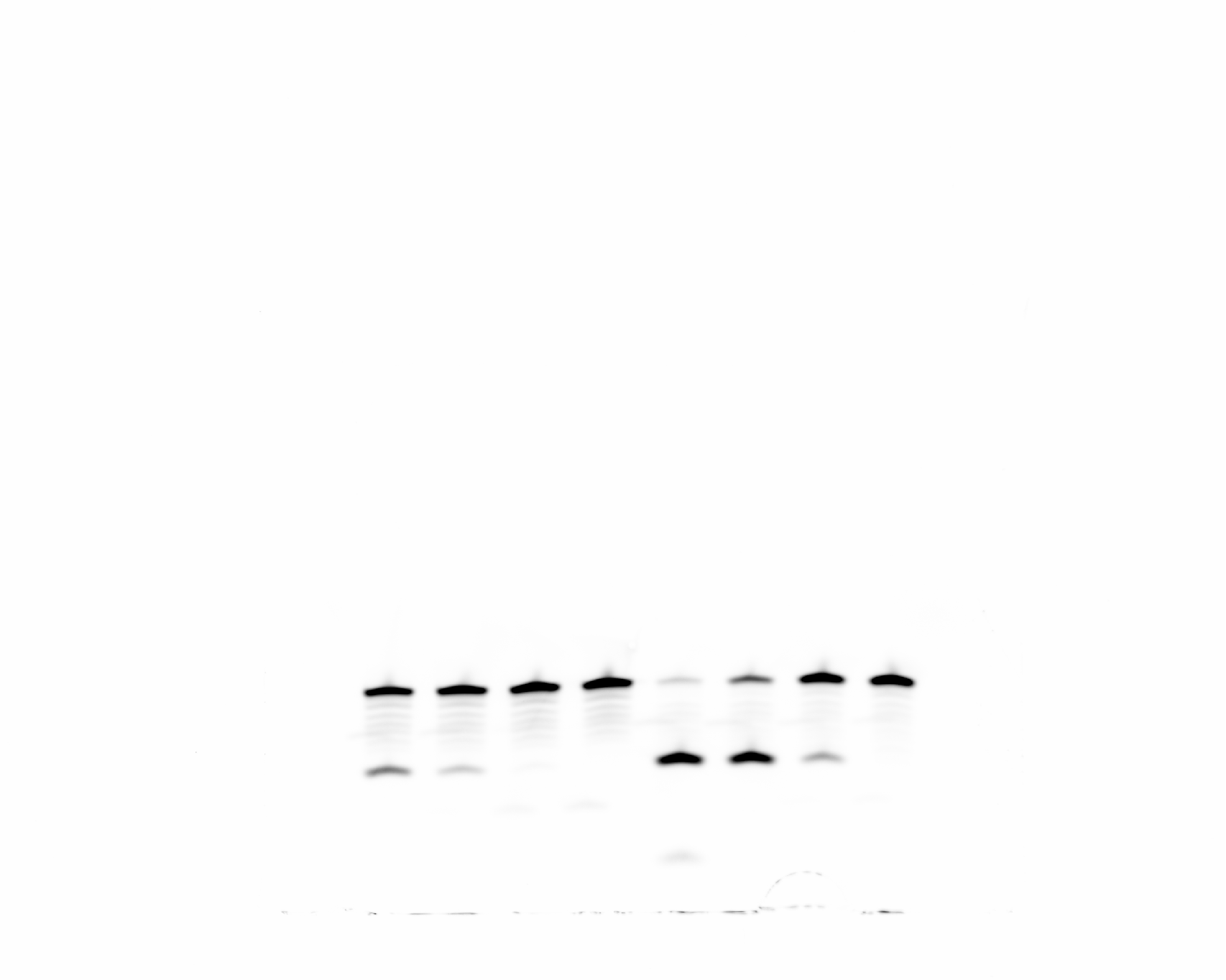

Supplement: Figure 4—source data 1. [file elife-90254-fig4-data1.zip › Figure 4C raw 1.tif]

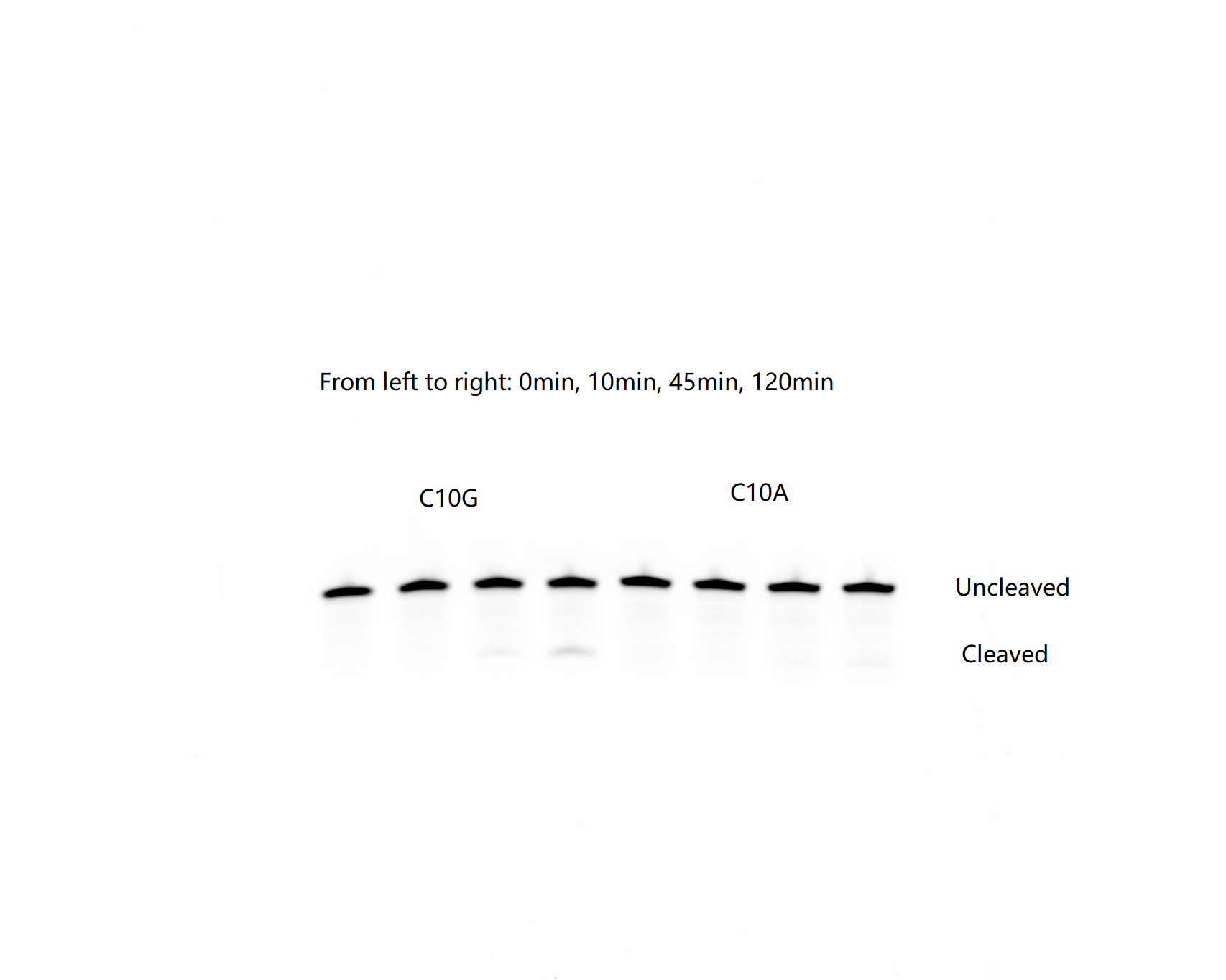

Supplement: Figure 4—source data 2. [file elife-90254-fig4-data2.zip › Figure 4C labeled 2.tif]

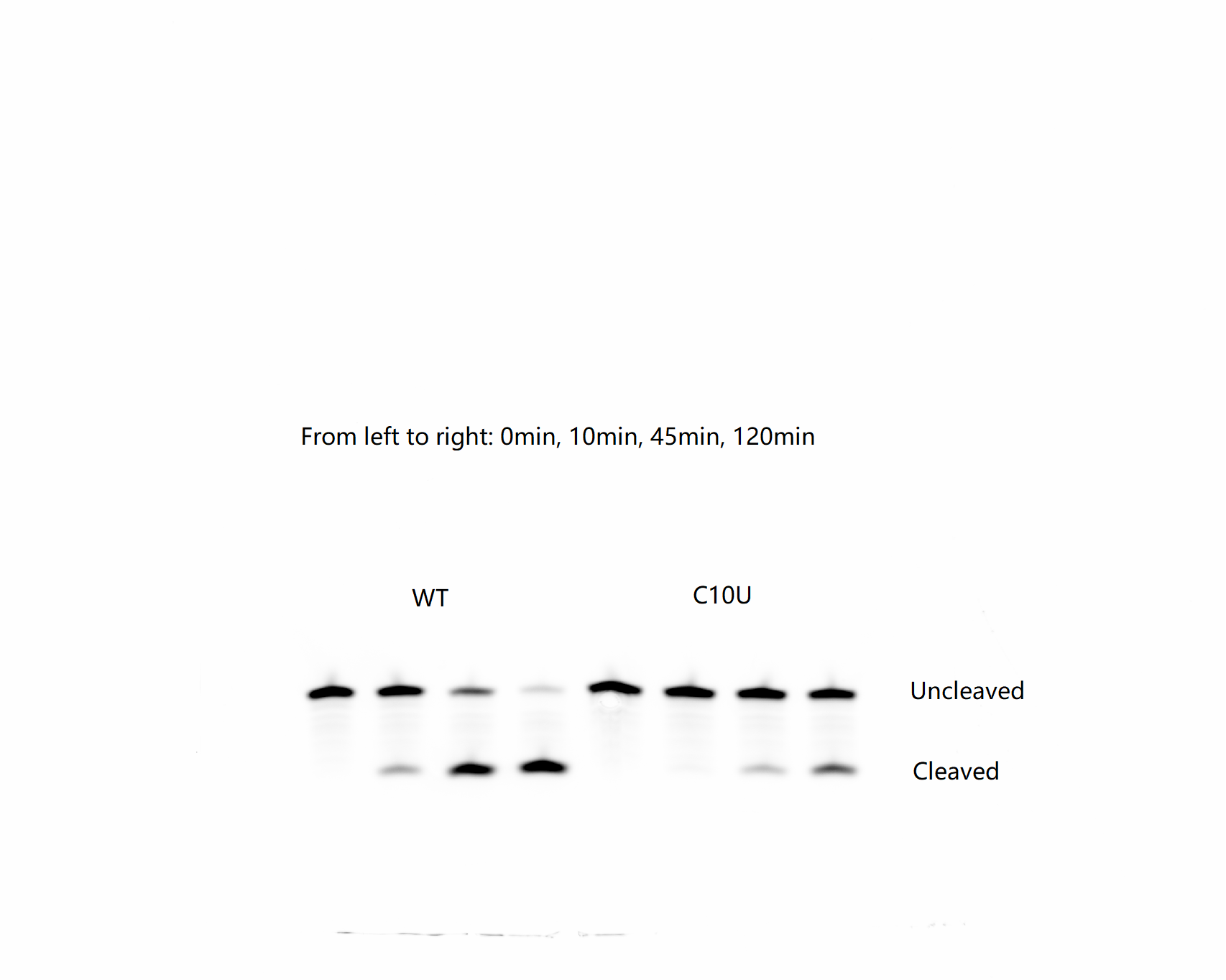

Supplement: Figure 4—source data 2. [file elife-90254-fig4-data2.zip › Figure 4C labeled 3.tif]

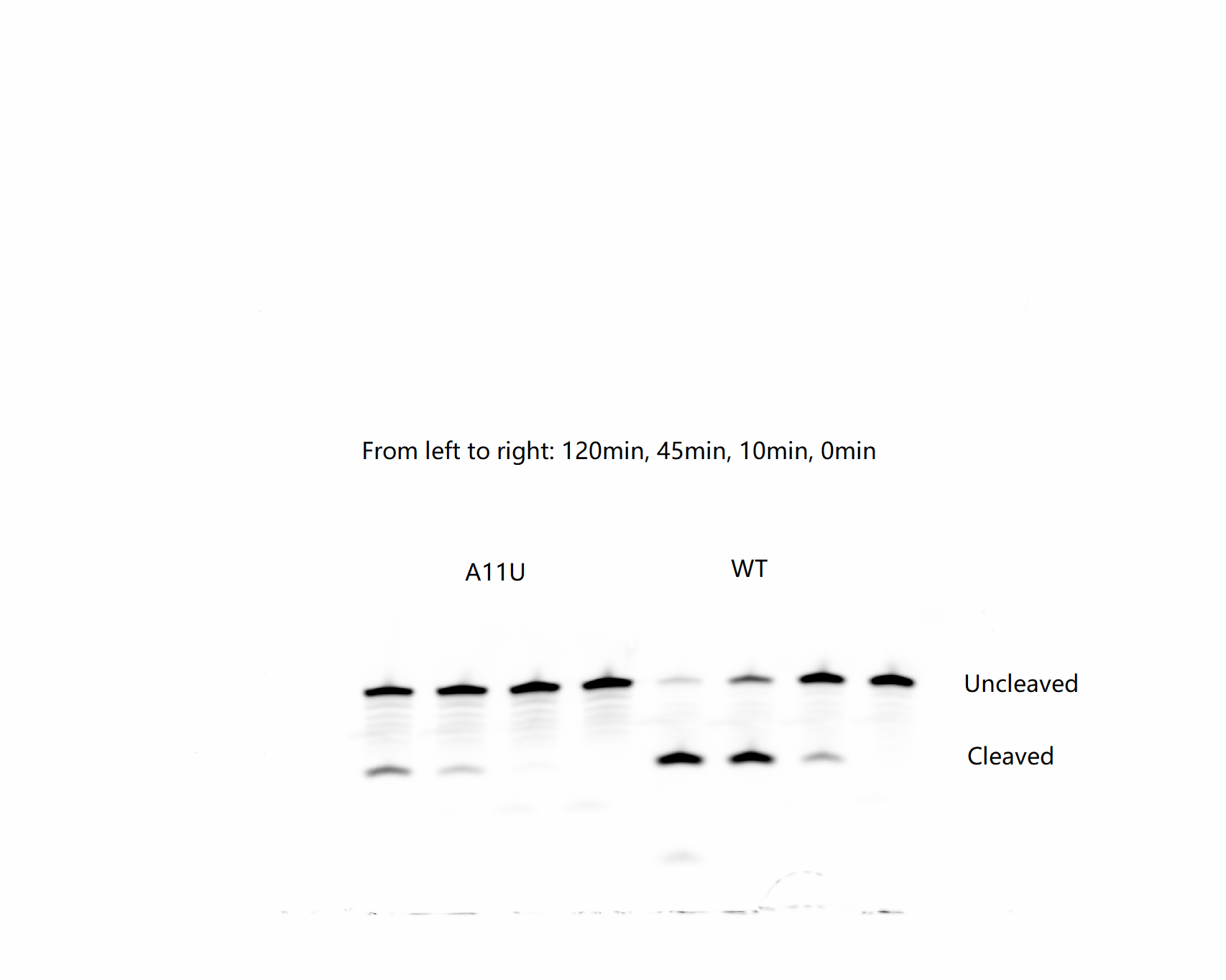

Supplement: Figure 4—source data 2. [file elife-90254-fig4-data2.zip › Figure 4C labeled 1.tif]

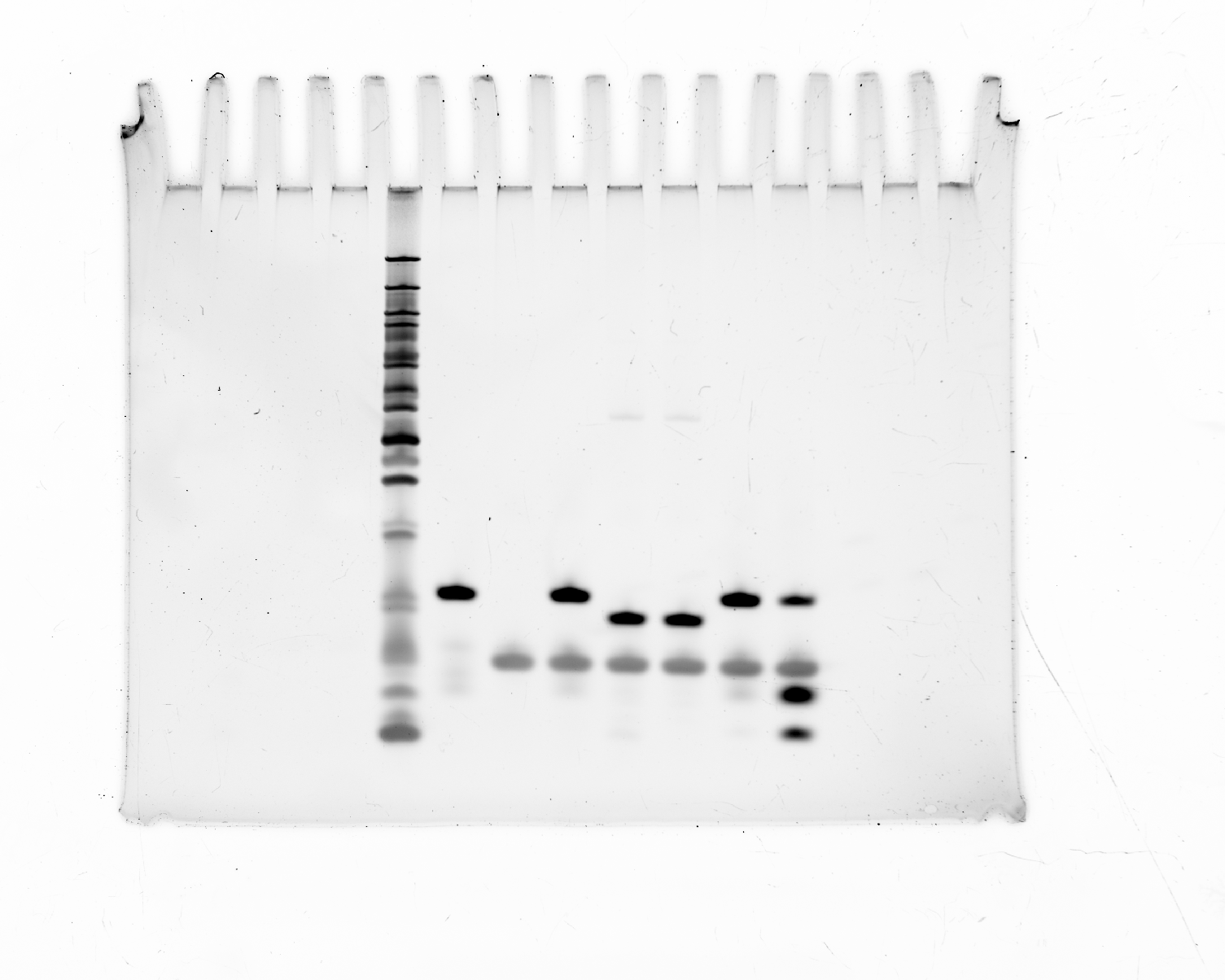

Supplement: Figure 5—source data 1. [file elife-90254-fig5-data1.zip › Figure 5B raw 2.tif]

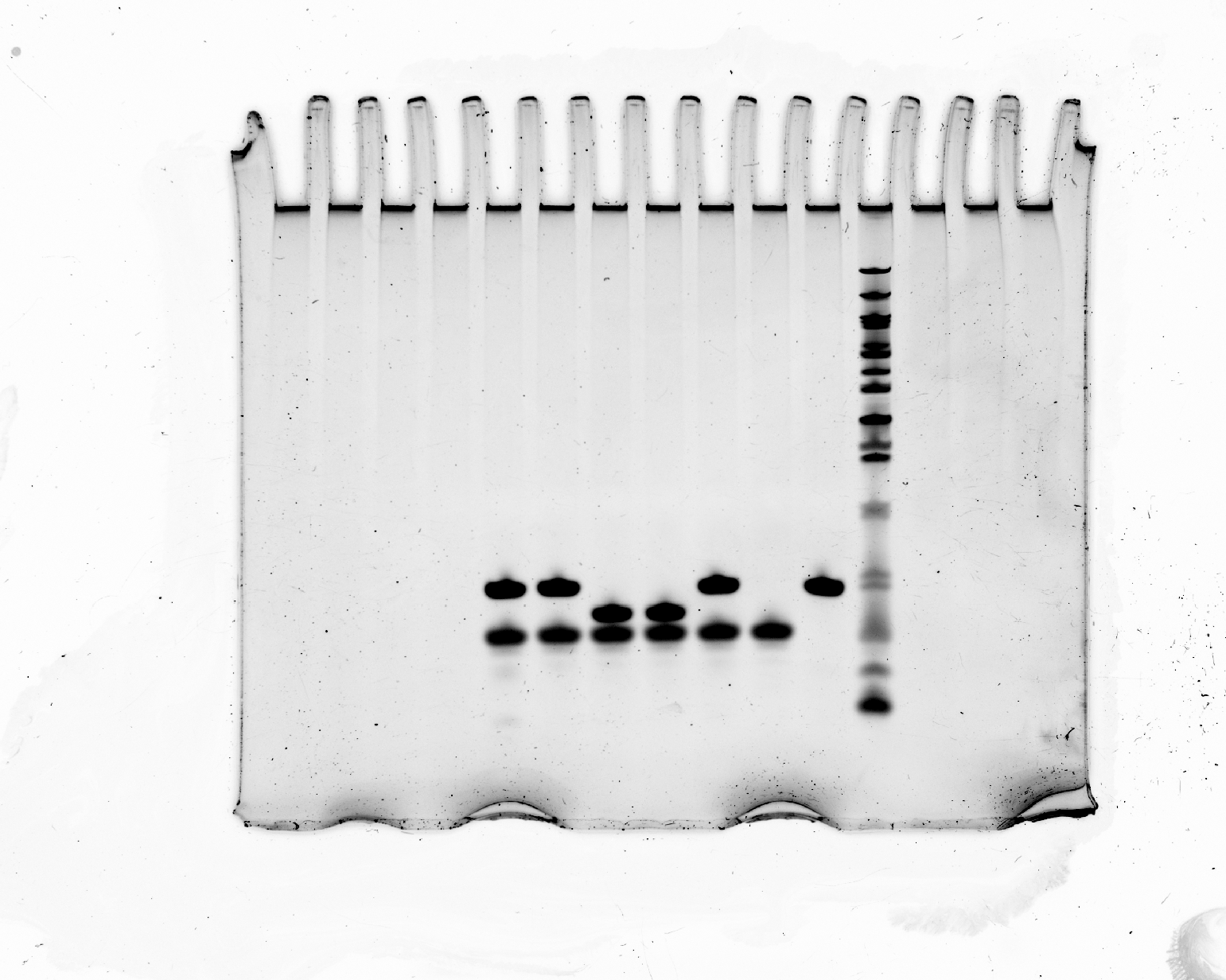

Supplement: Figure 5—source data 1. [file elife-90254-fig5-data1.zip › Figure 5B raw 3.tif]

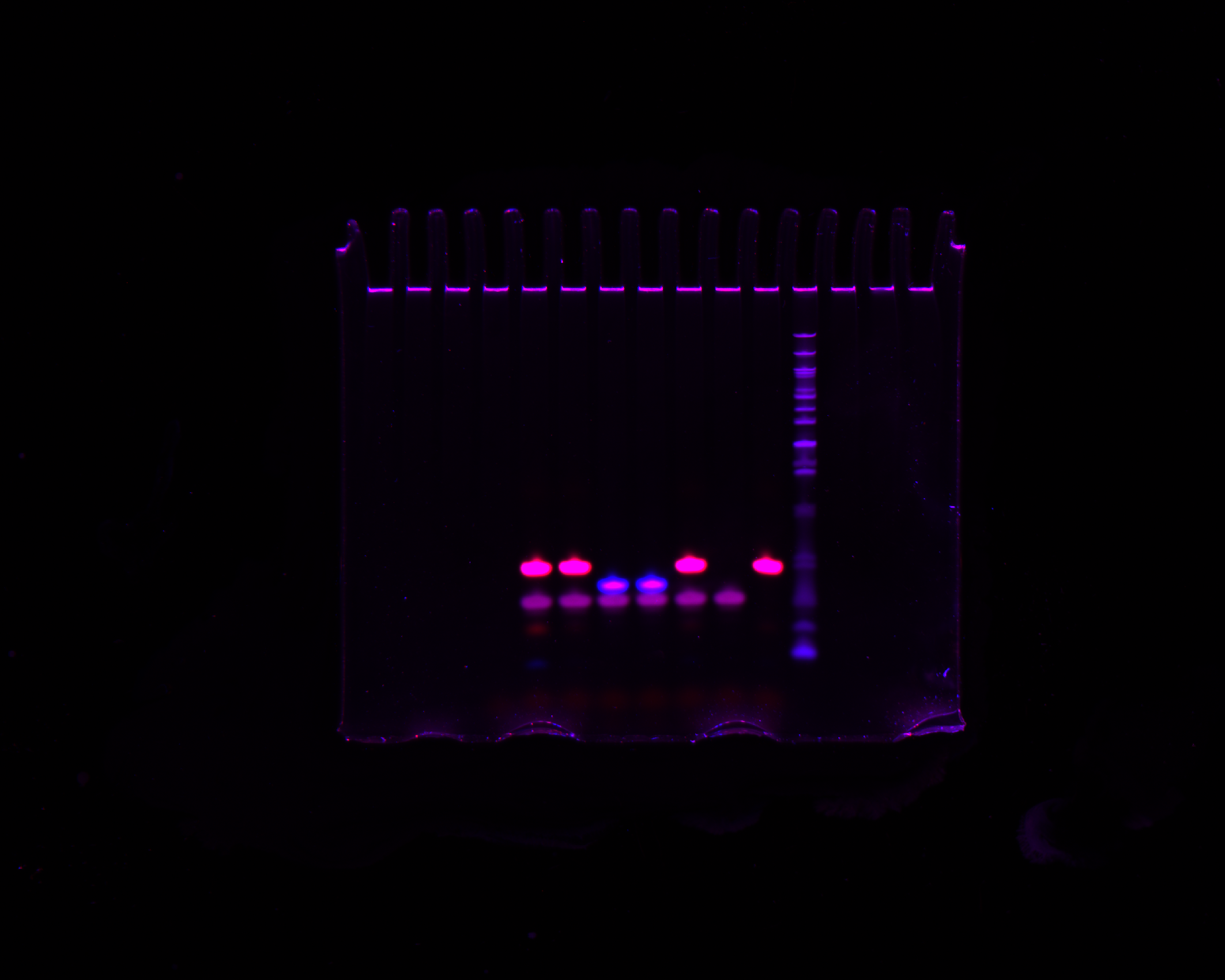

Supplement: Figure 5—source data 1. [file elife-90254-fig5-data1.zip › Figure 5B raw 4.tif]

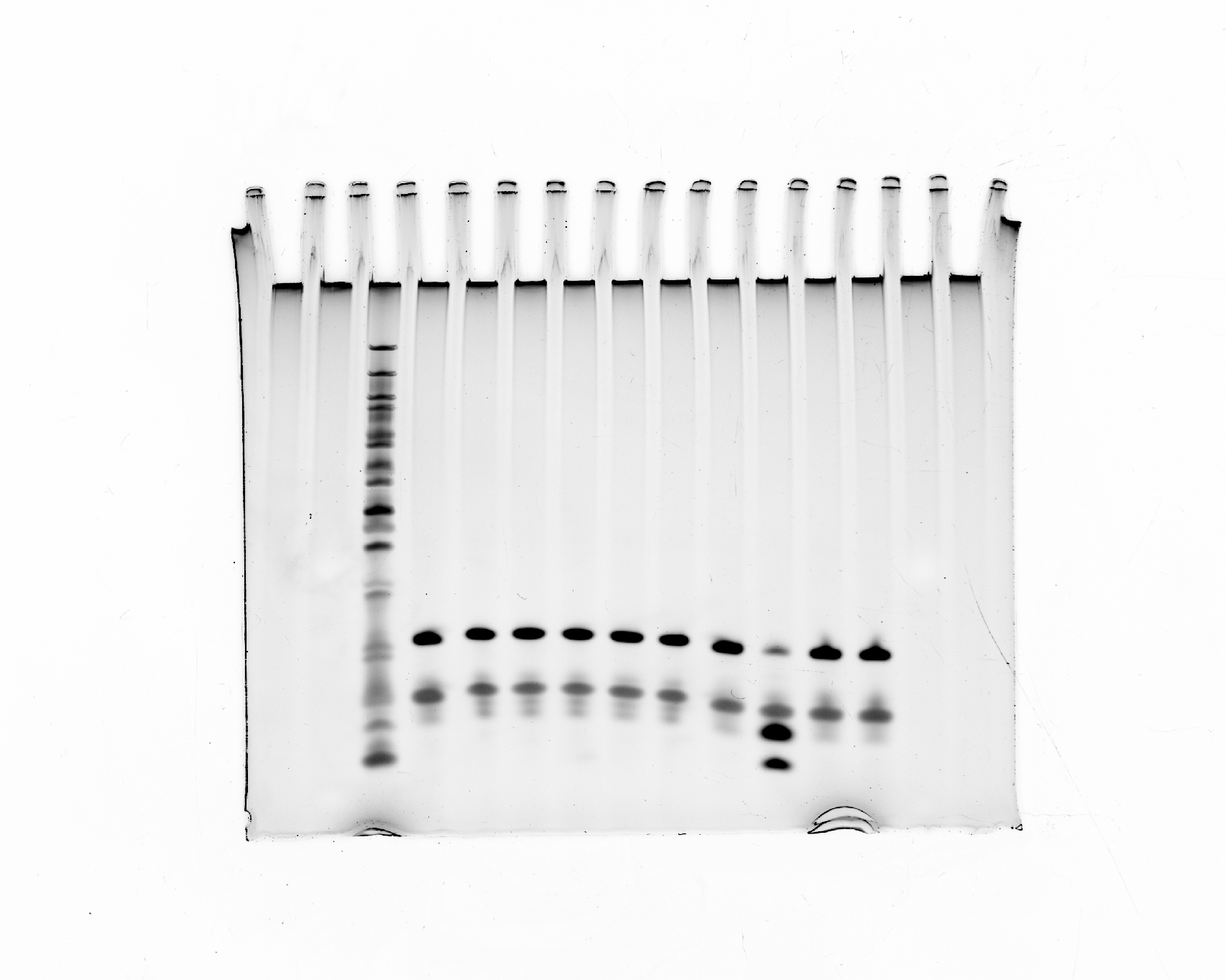

Supplement: Figure 5—source data 1. [file elife-90254-fig5-data1.zip › Figure 5CD raw 5.tif]

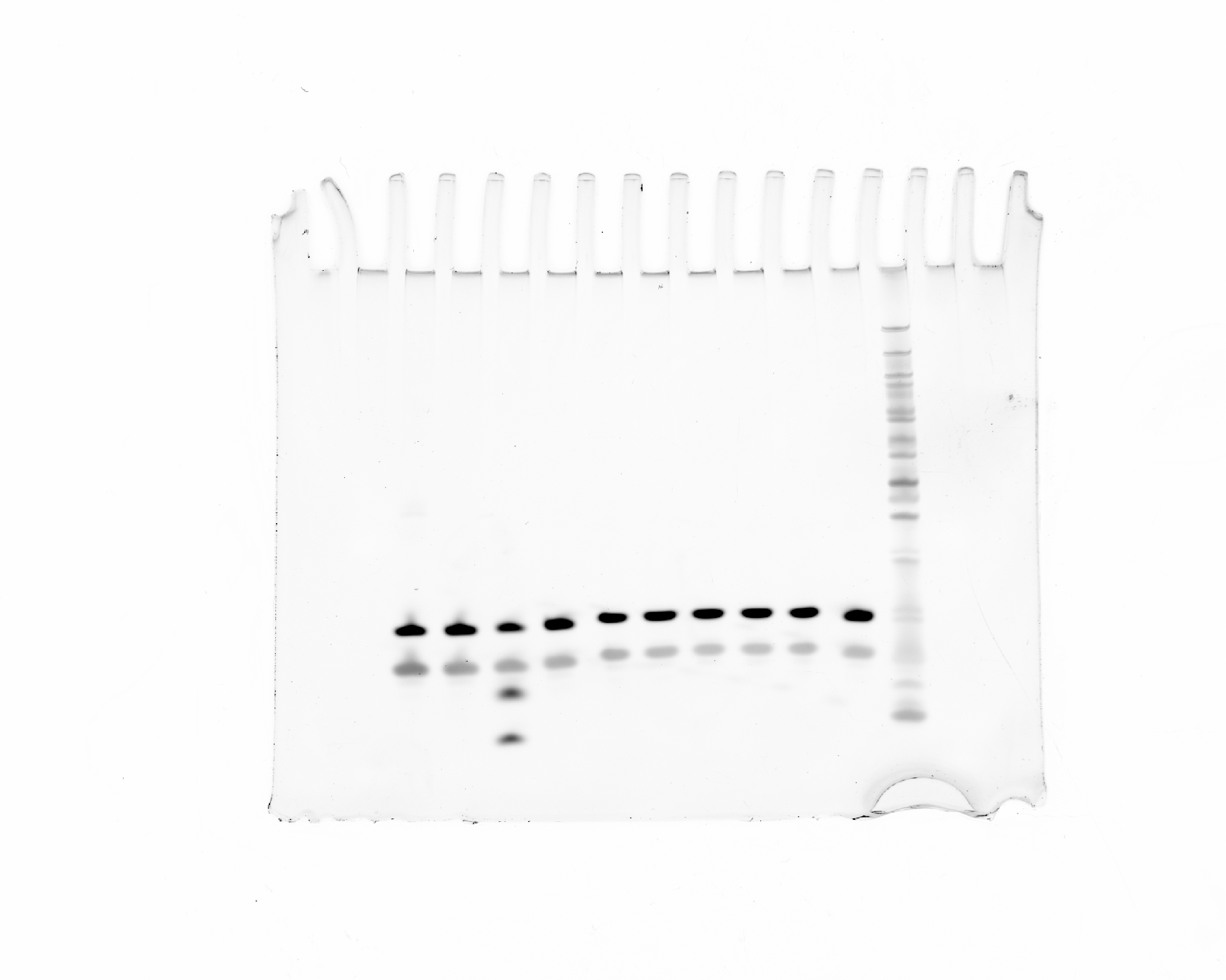

Supplement: Figure 5—source data 1. [file elife-90254-fig5-data1.zip › Figure 5CD raw 6.tif]

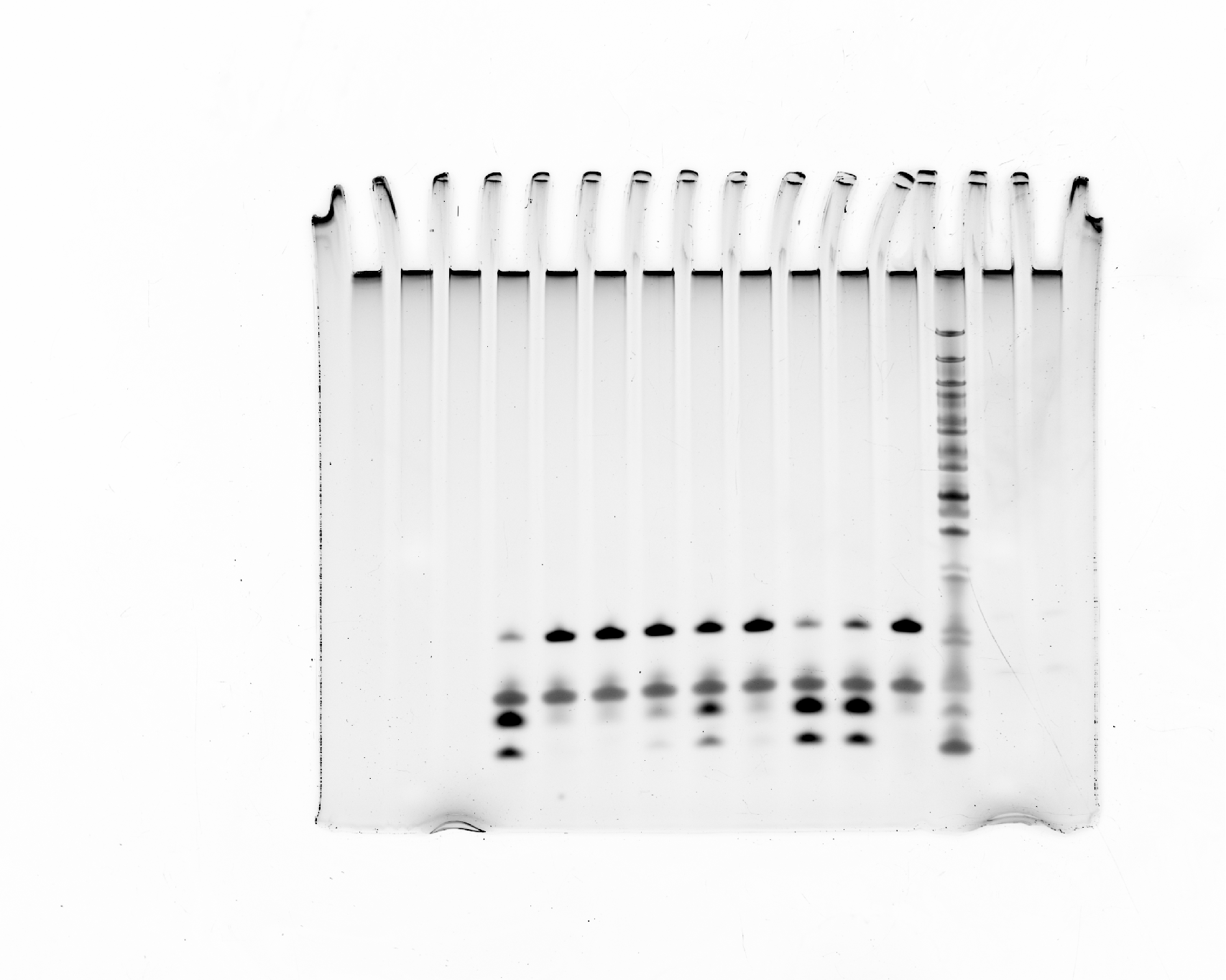

Supplement: Figure 5—source data 1. [file elife-90254-fig5-data1.zip › Figure 5D raw 7.tif]

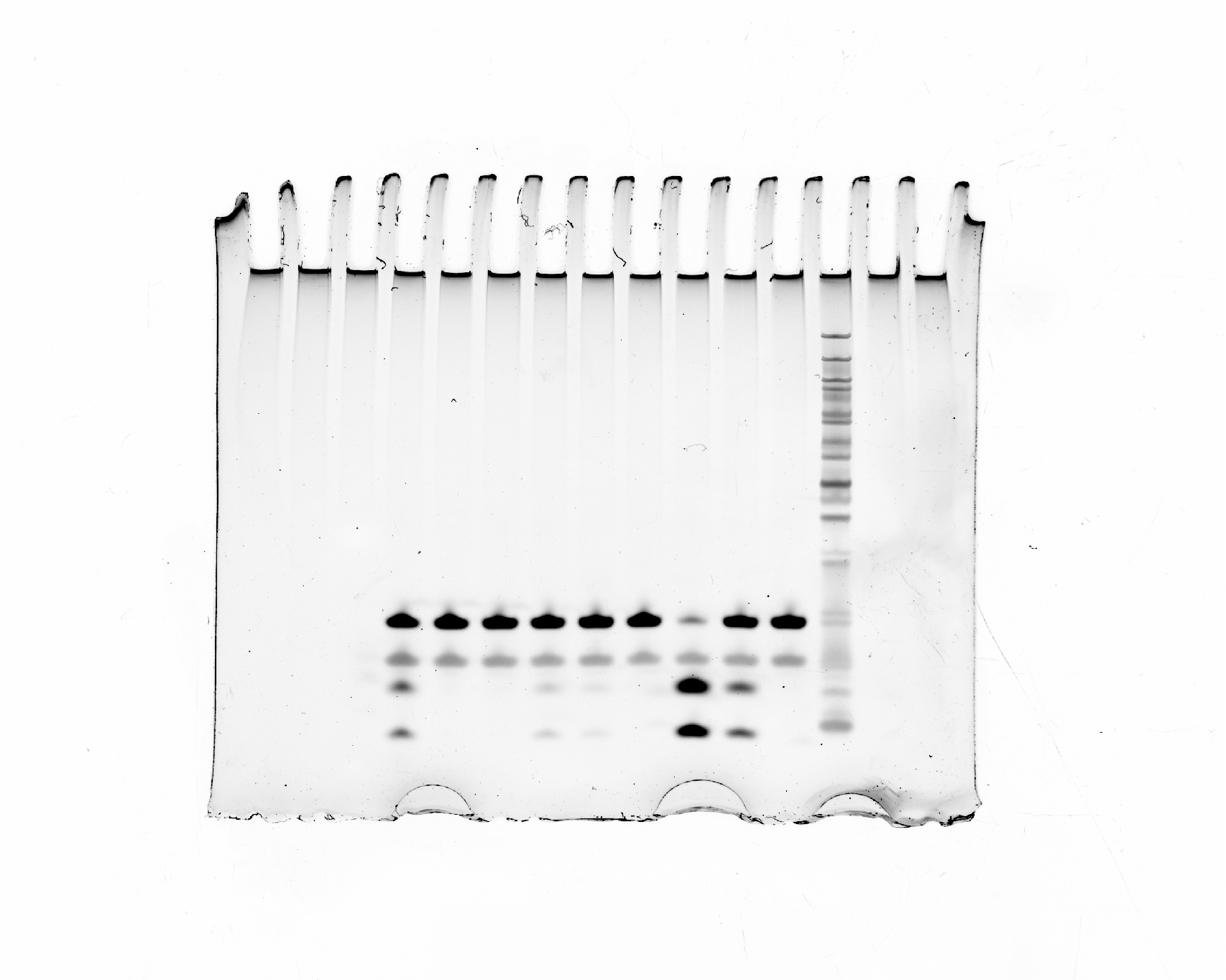

Supplement: Figure 5—source data 1. [file elife-90254-fig5-data1.zip › Figure 5D raw 8.tif]

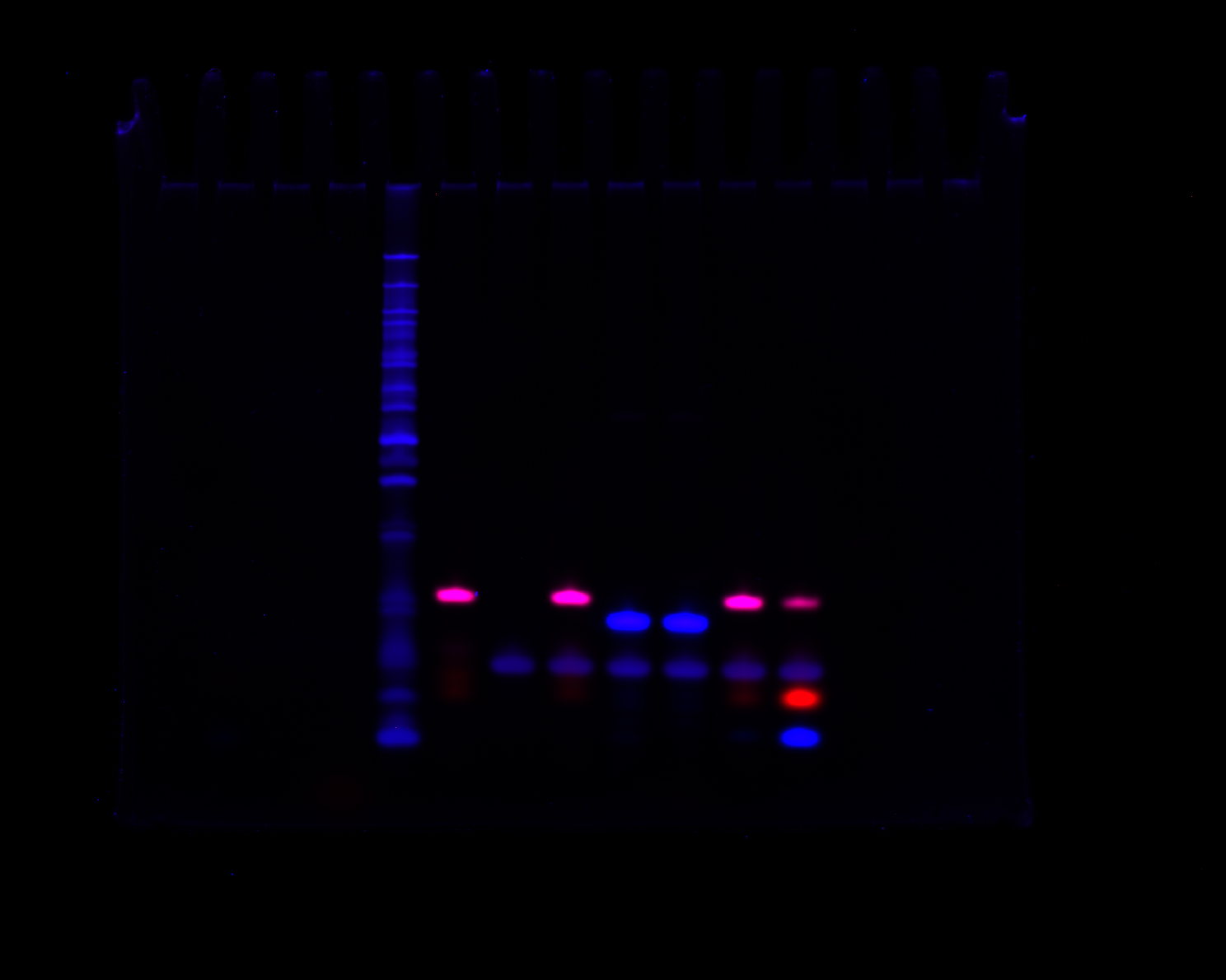

Supplement: Figure 5—source data 1. [file elife-90254-fig5-data1.zip › Figure 5B raw 1.tif]

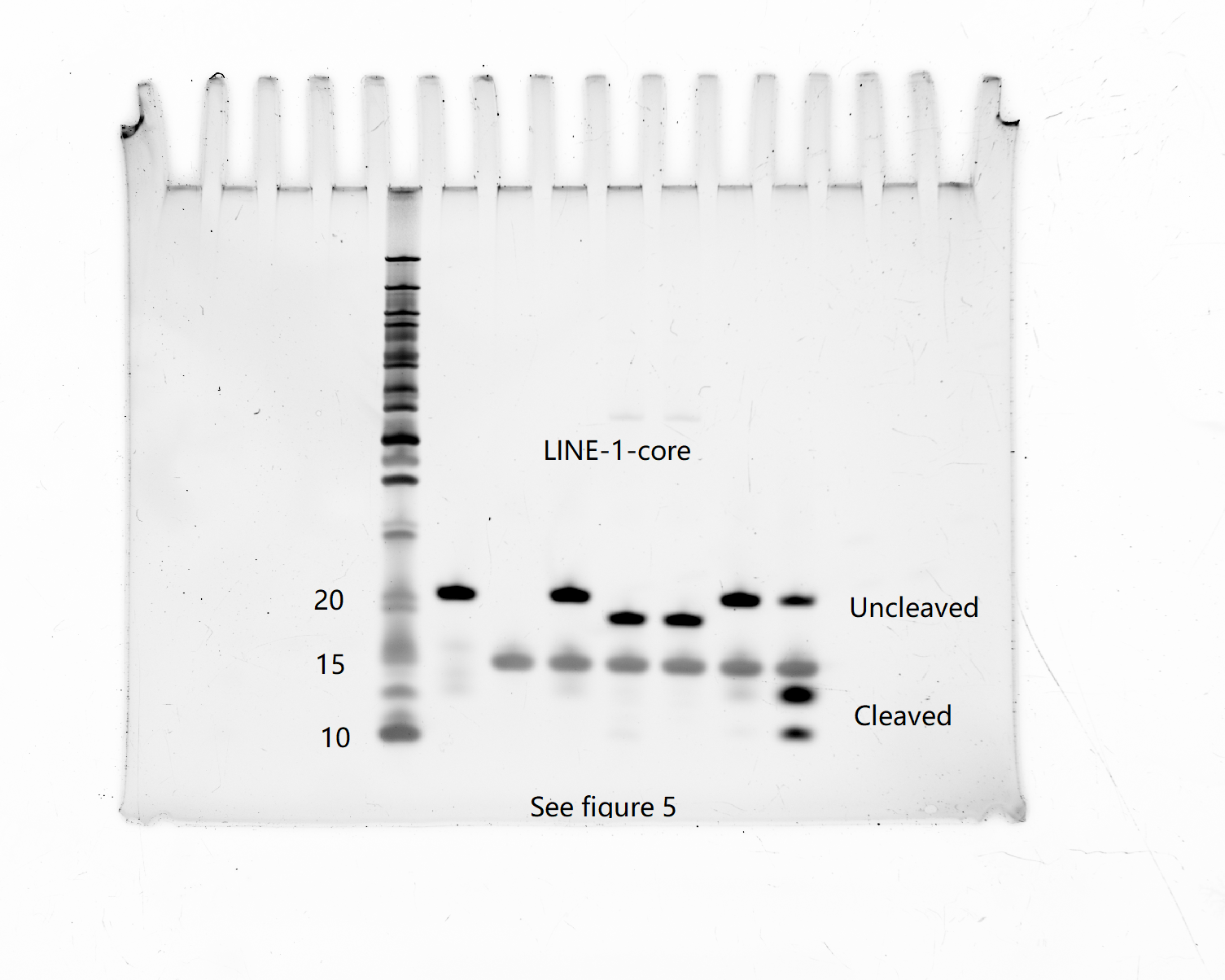

Supplement: Figure 5—source data 2. [file elife-90254-fig5-data2.zip › Figure 5B labeled 2.tif]

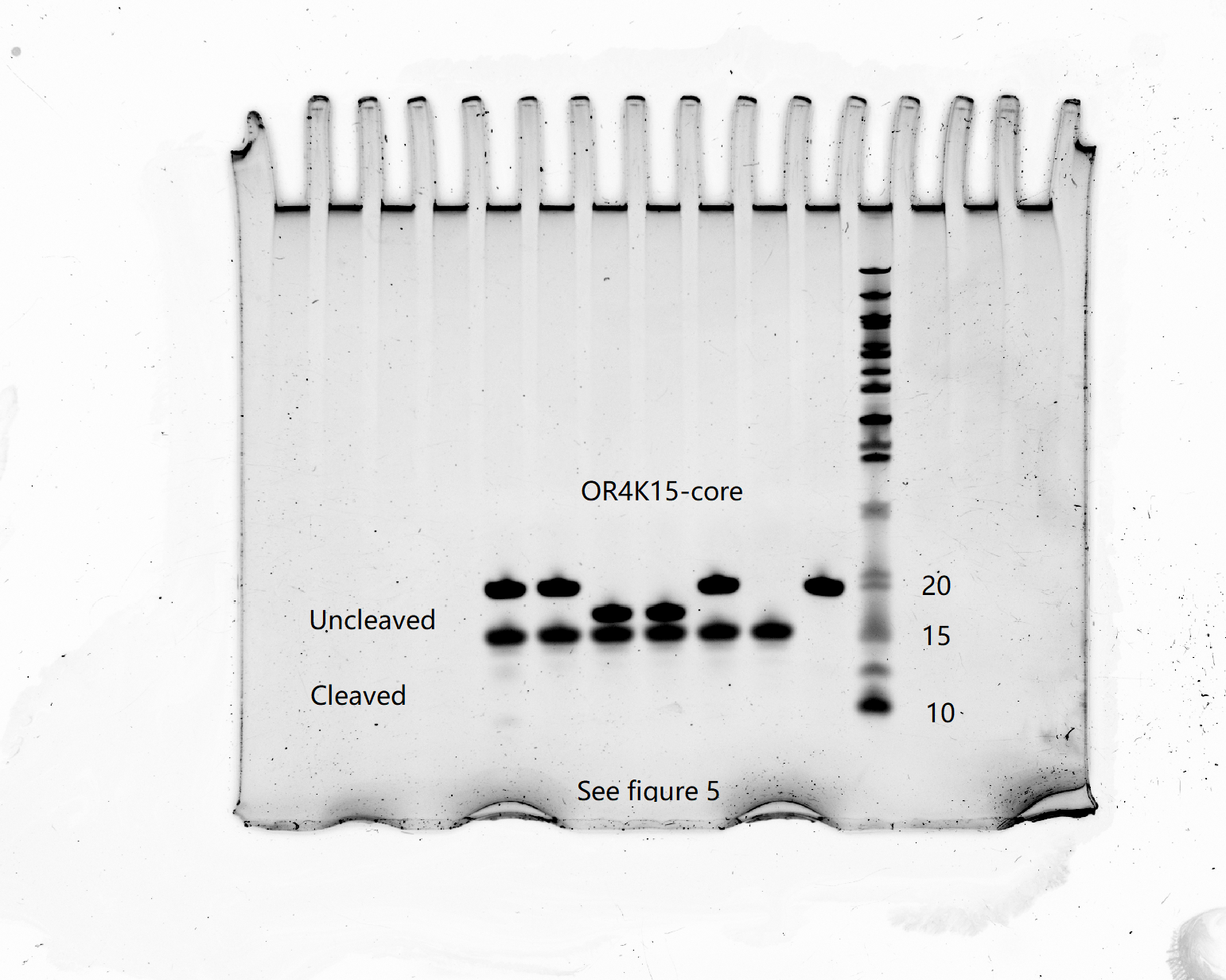

Supplement: Figure 5—source data 2. [file elife-90254-fig5-data2.zip › Figure 5B labeled 3.tif]

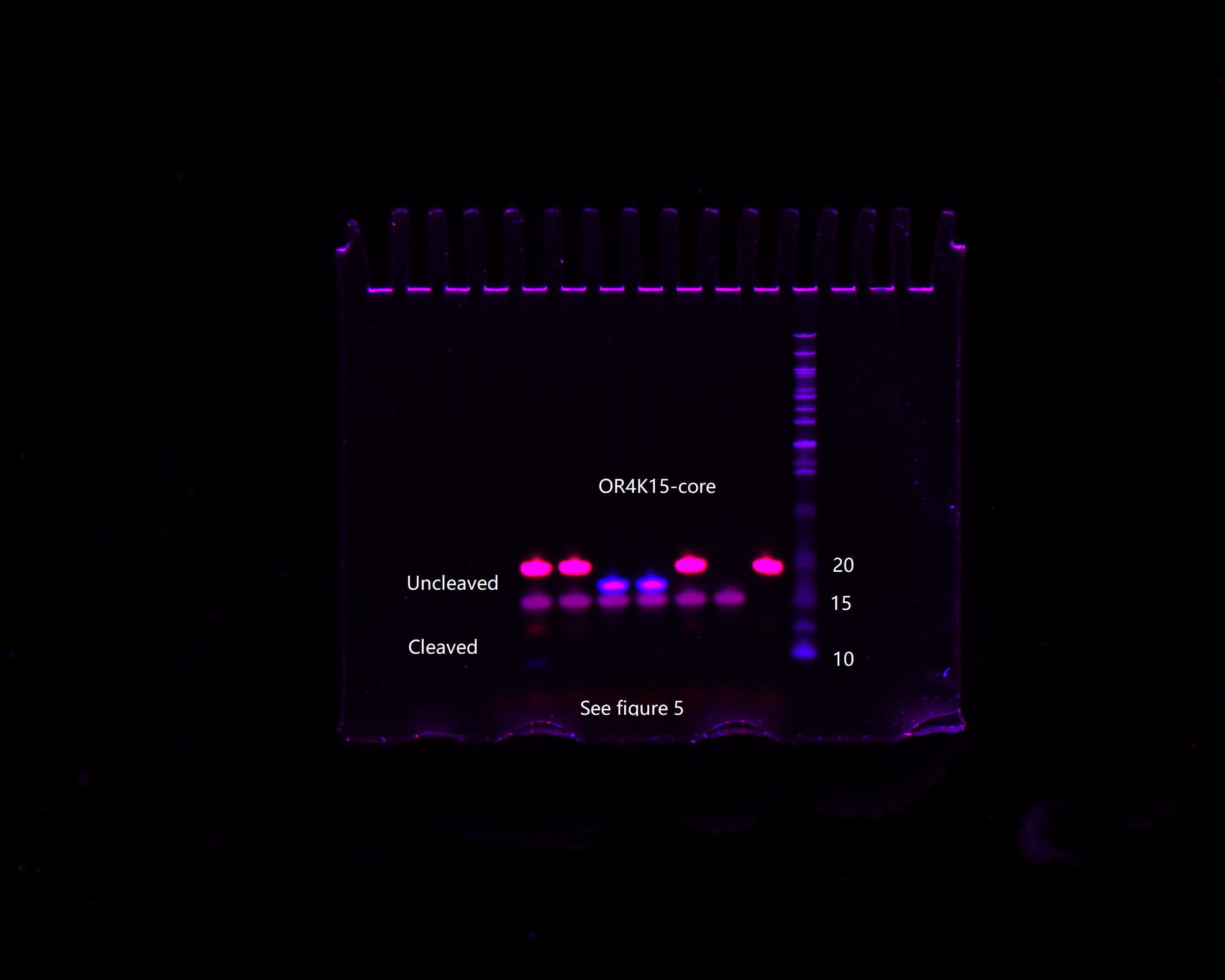

Supplement: Figure 5—source data 2. [file elife-90254-fig5-data2.zip › Figure 5B labeled 4.tif]

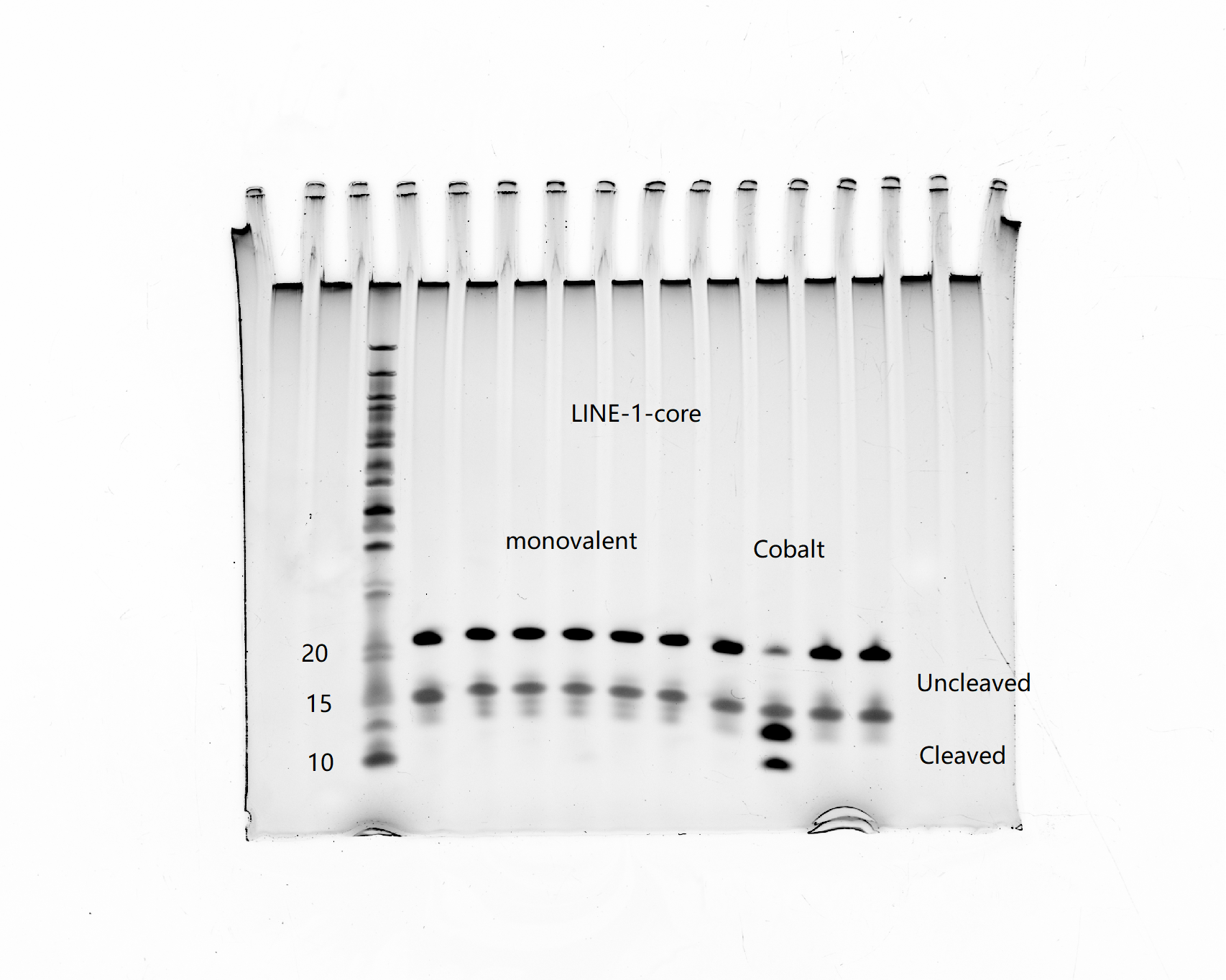

Supplement: Figure 5—source data 2. [file elife-90254-fig5-data2.zip › Figure 5CD labeled 5.tif]

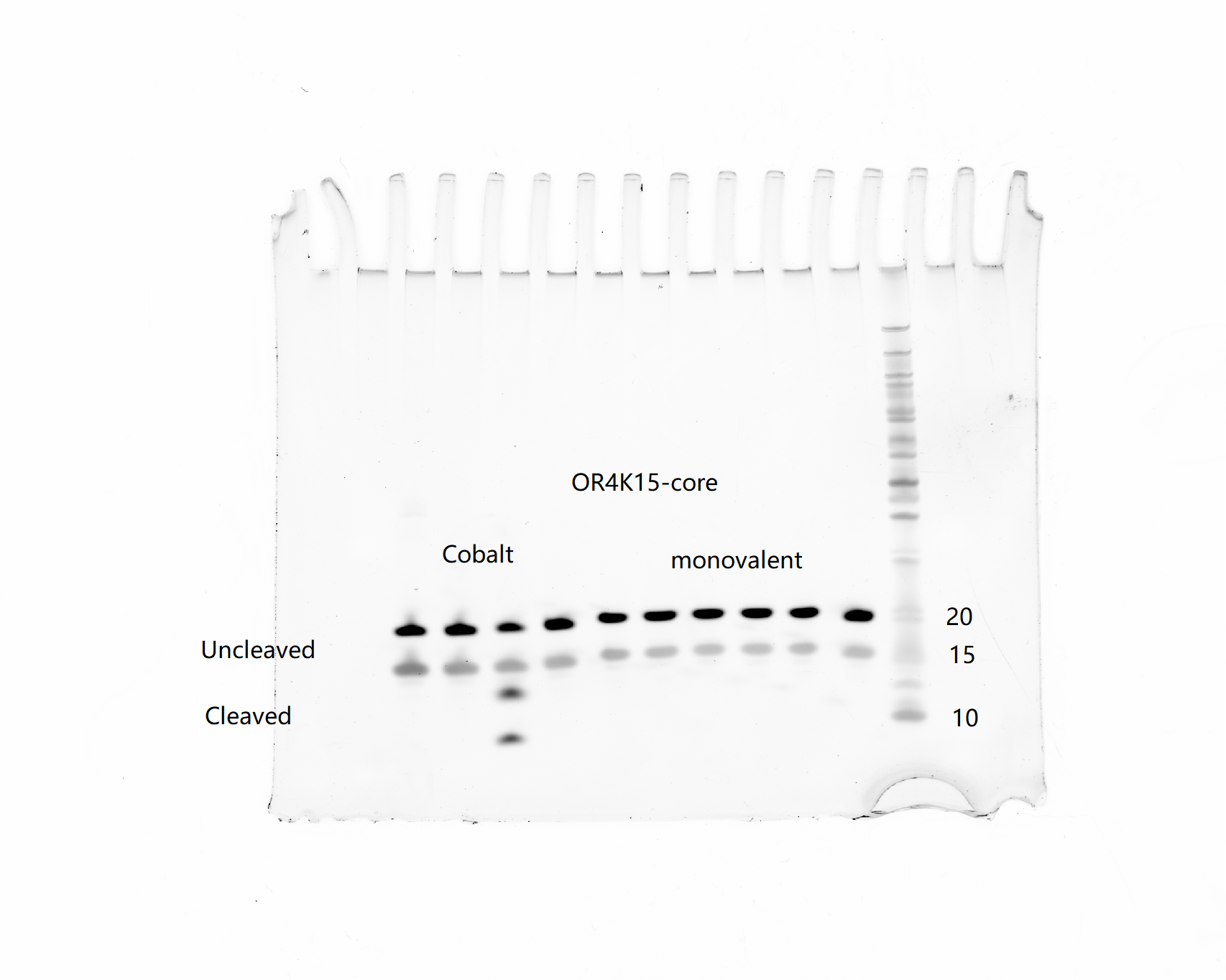

Supplement: Figure 5—source data 2. [file elife-90254-fig5-data2.zip › Figure 5CD labeled 6.tif]

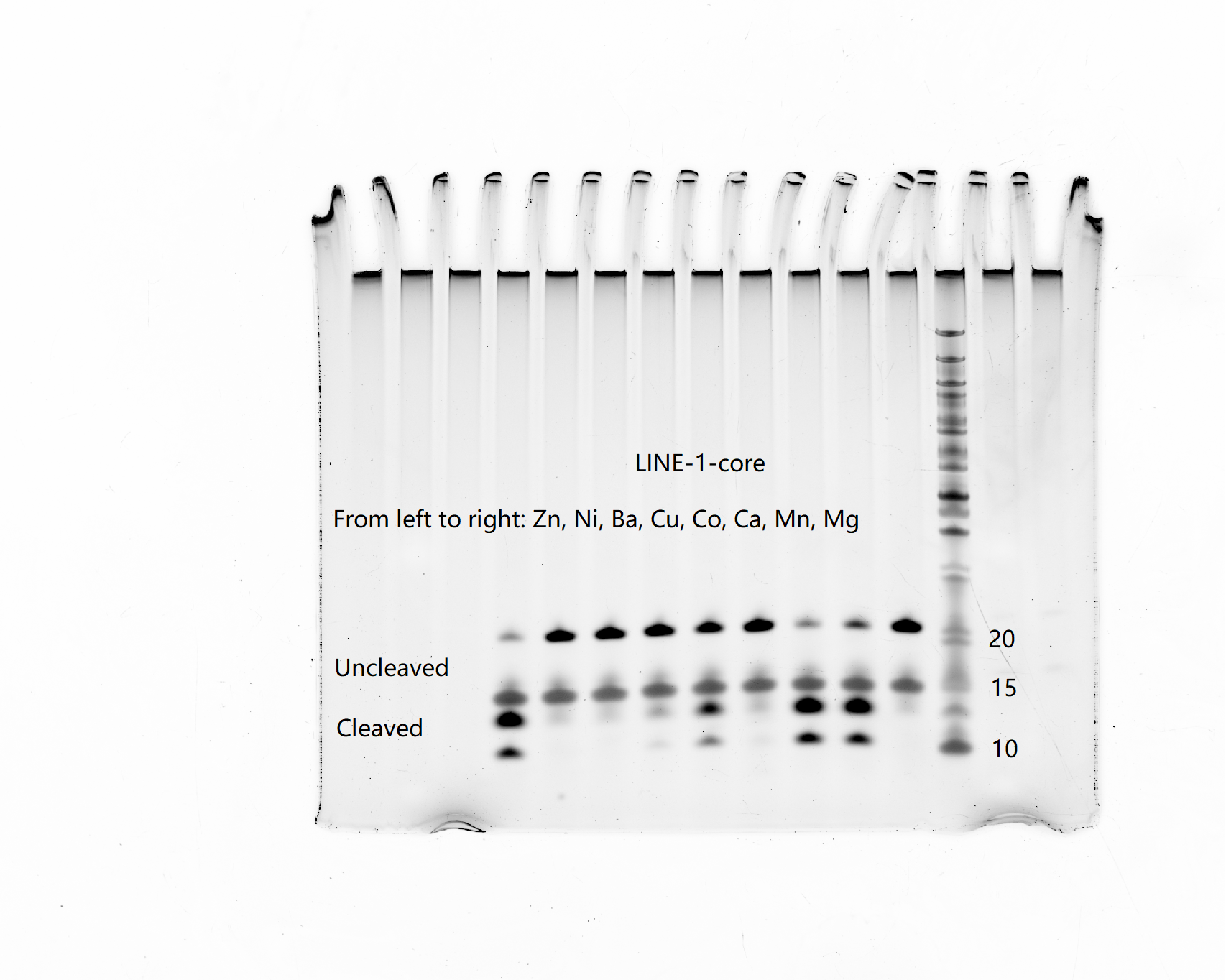

Supplement: Figure 5—source data 2. [file elife-90254-fig5-data2.zip › Figure 5D labeled 7.tif]

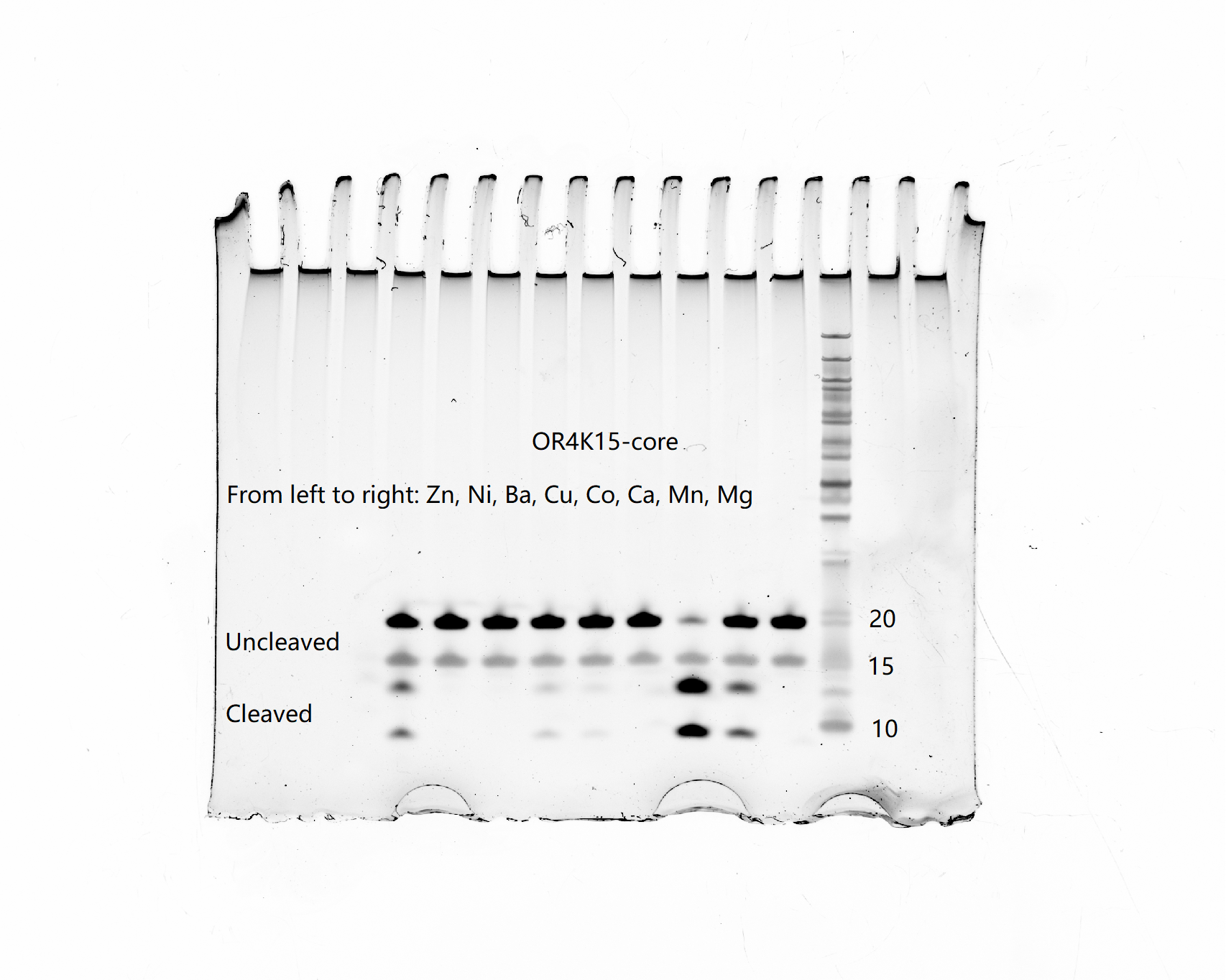

Supplement: Figure 5—source data 2. [file elife-90254-fig5-data2.zip › Figure 5D labeled 8.tif]

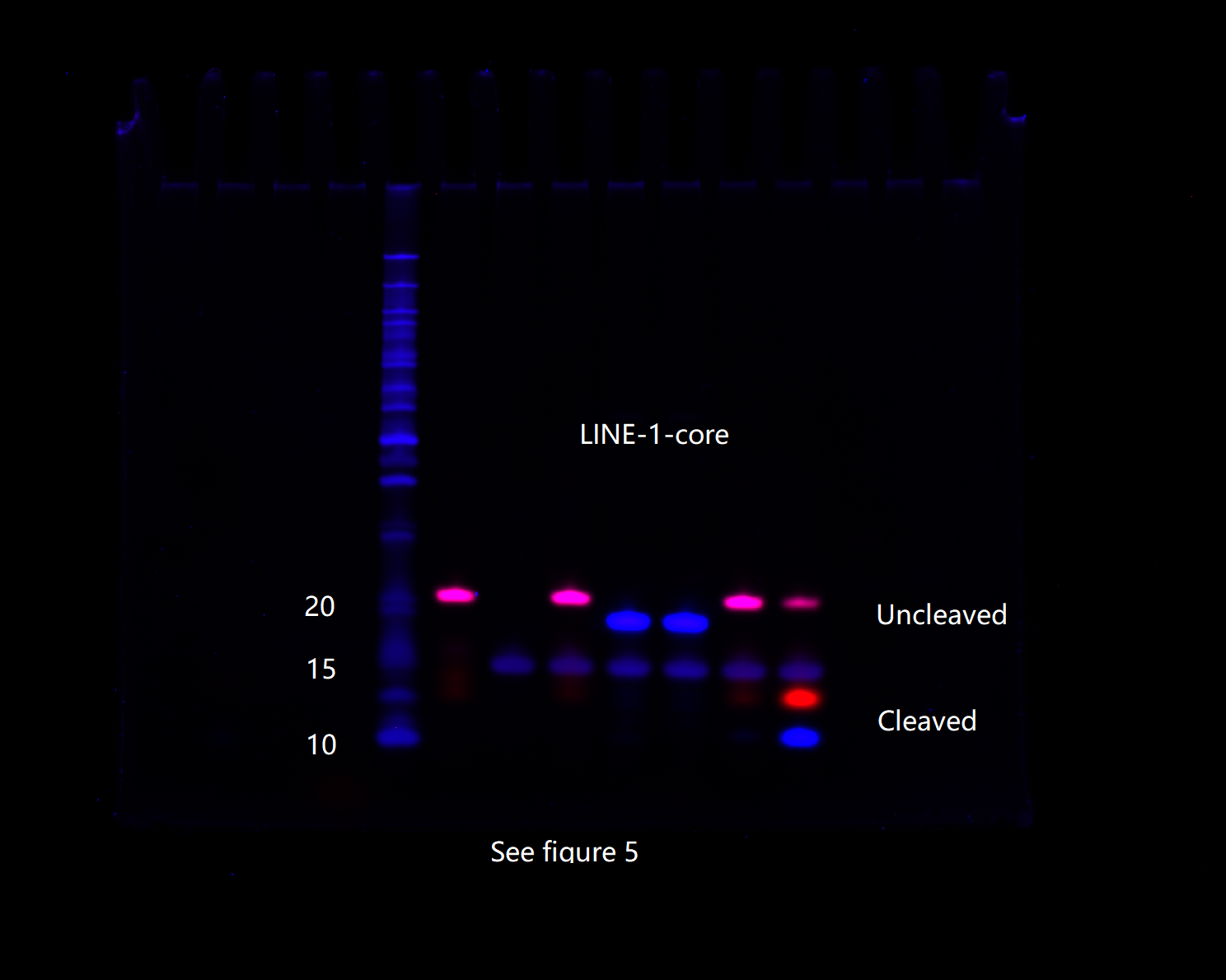

Supplement: Figure 5—source data 2. [file elife-90254-fig5-data2.zip › Figure 5B labeled 1.tif]

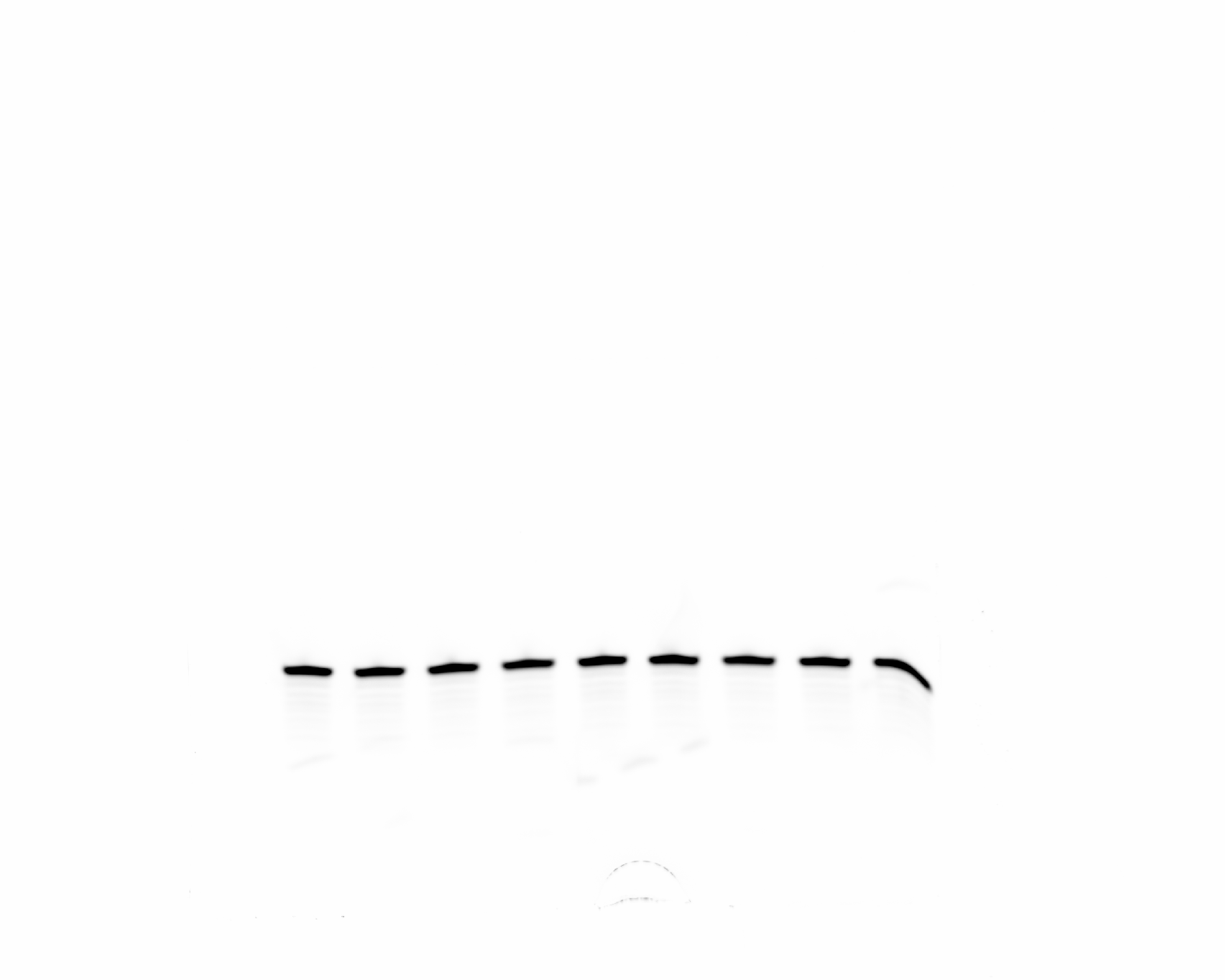

Supplement: Figure 5—figure supplement 1—source data 1. [file elife-90254-fig5-figsupp1-data1.zip › Figure 5-figure supplement 1 raw 2.tif]

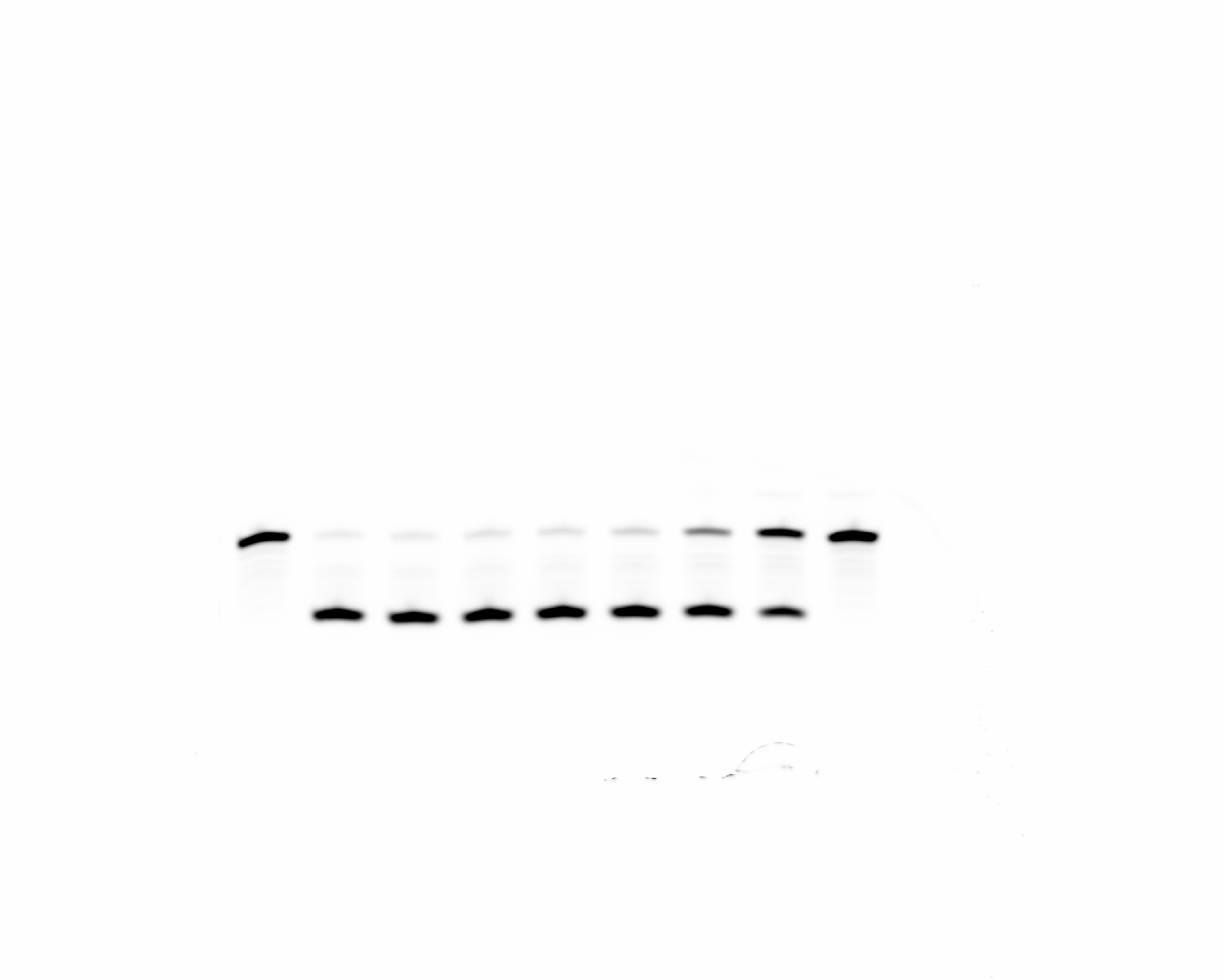

Supplement: Figure 5—figure supplement 1—source data 1. [file elife-90254-fig5-figsupp1-data1.zip › Figure 5-figure supplement 1 raw 1.tif]

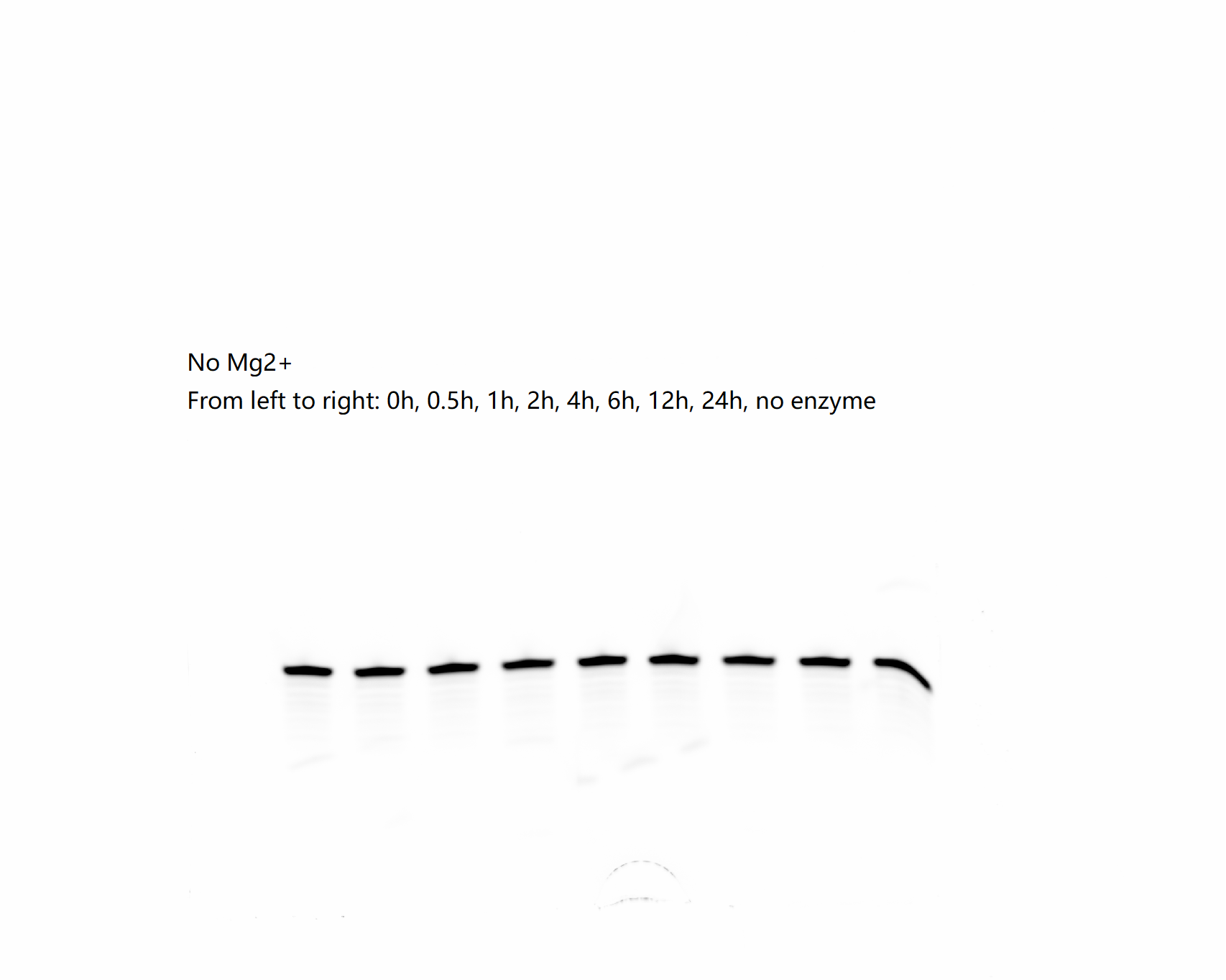

Supplement: Figure 5—figure supplement 1—source data 2. [file elife-90254-fig5-figsupp1-data2.zip › Figure 5-figure supplement 1 labeled 2.tif]

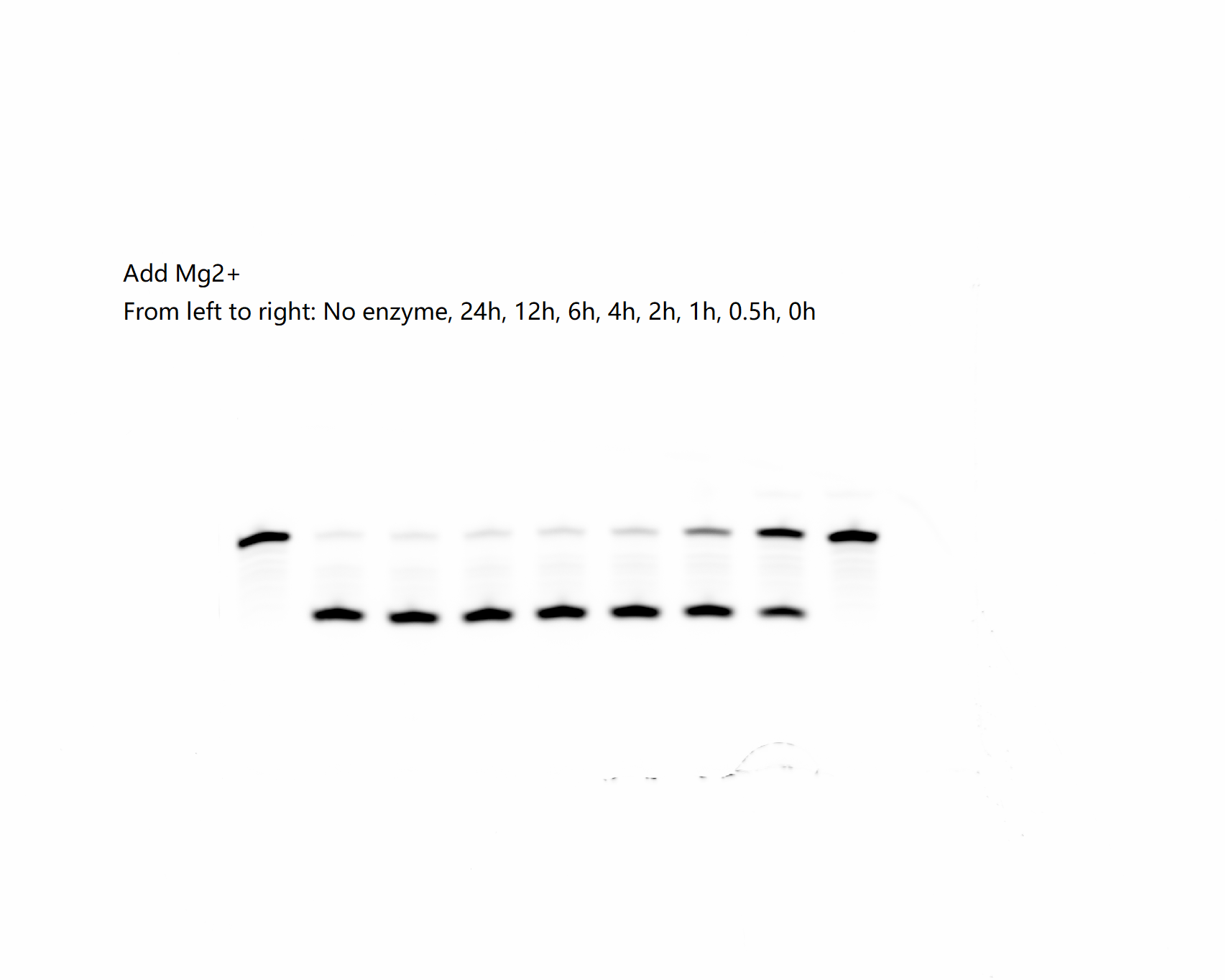

Supplement: Figure 5—figure supplement 1—source data 2. [file elife-90254-fig5-figsupp1-data2.zip › Figure 5-figure supplement 1 labeled 1.tif]

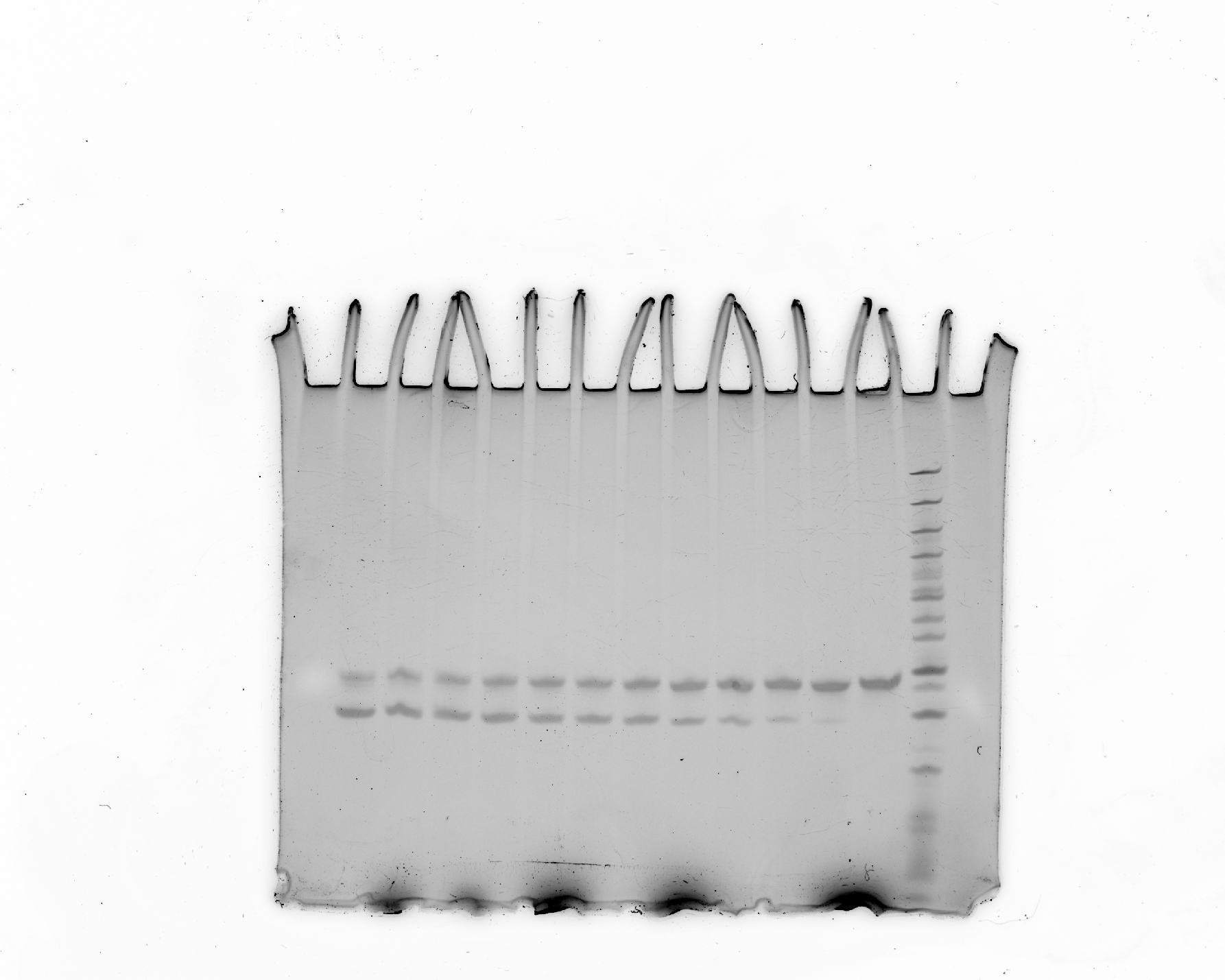

Supplement: Figure 5—figure supplement 5—source data 1. [file elife-90254-fig5-figsupp5-data1.zip › Figure 5-figure supplement 5 raw 2.tif]

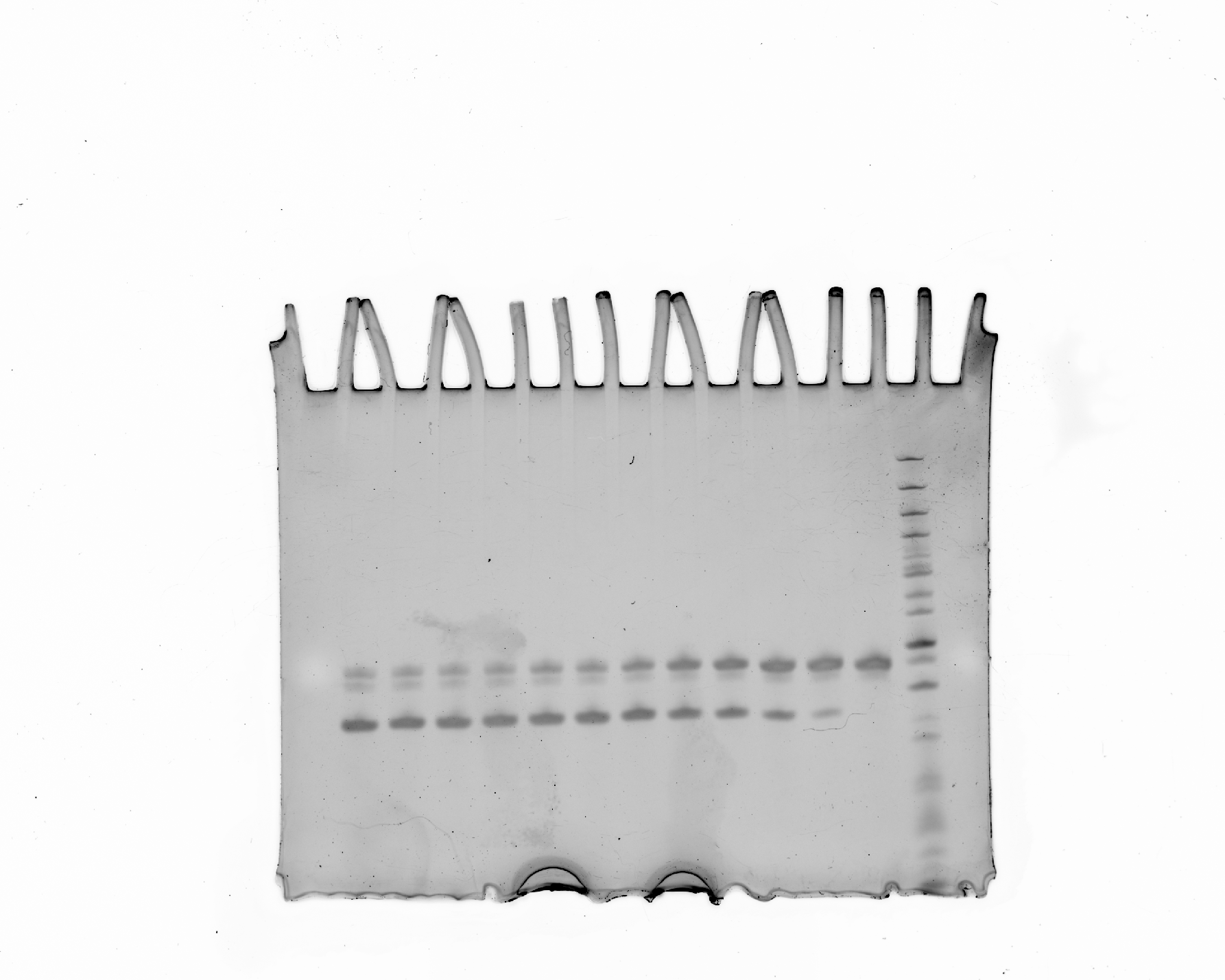

Supplement: Figure 5—figure supplement 5—source data 1. [file elife-90254-fig5-figsupp5-data1.zip › Figure 5-figure supplement 5 raw 1.tif]

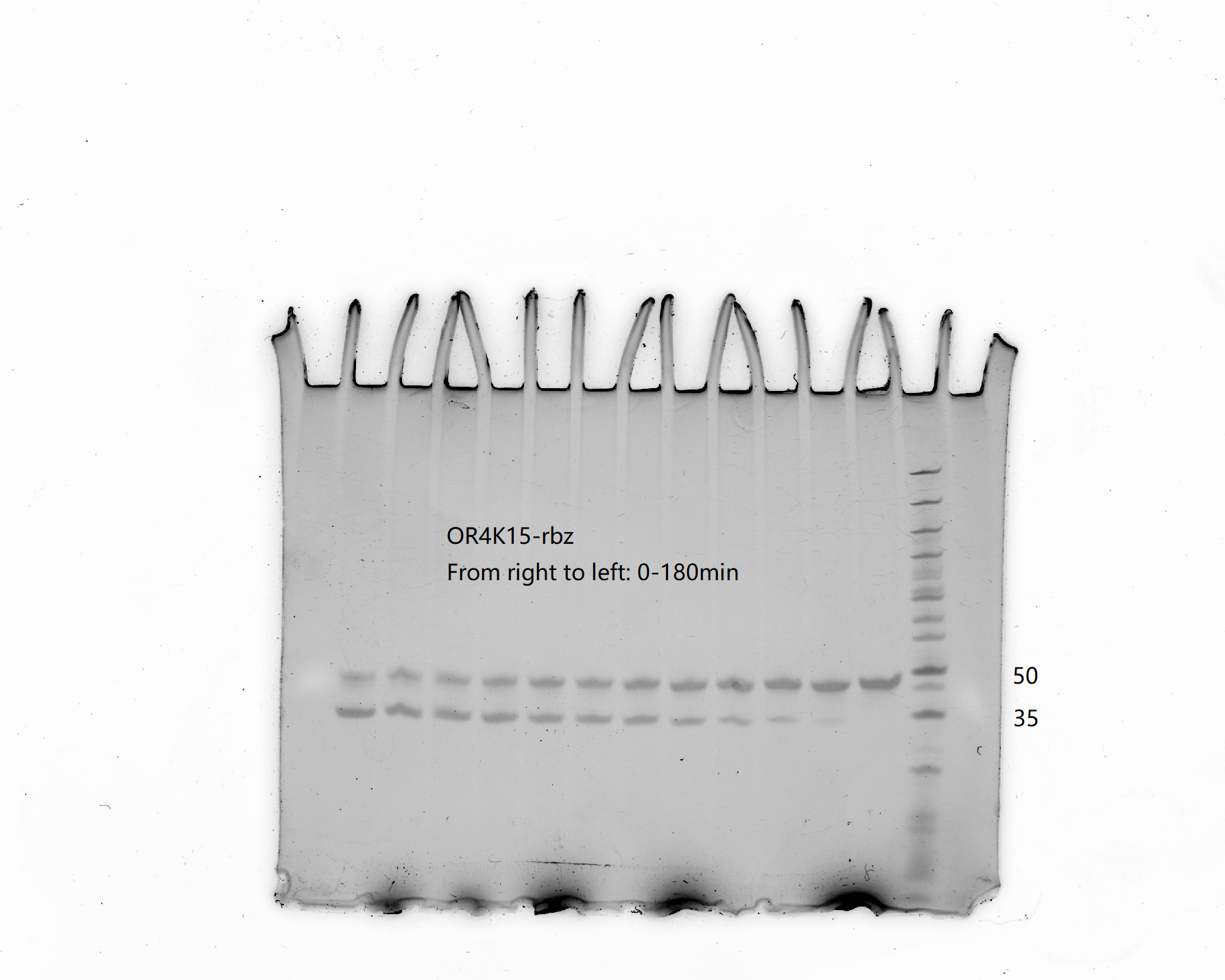

Supplement: Figure 5—figure supplement 5—source data 2. [file elife-90254-fig5-figsupp5-data2.zip › Figure 5-figure supplement 5 labeled 2.tif]

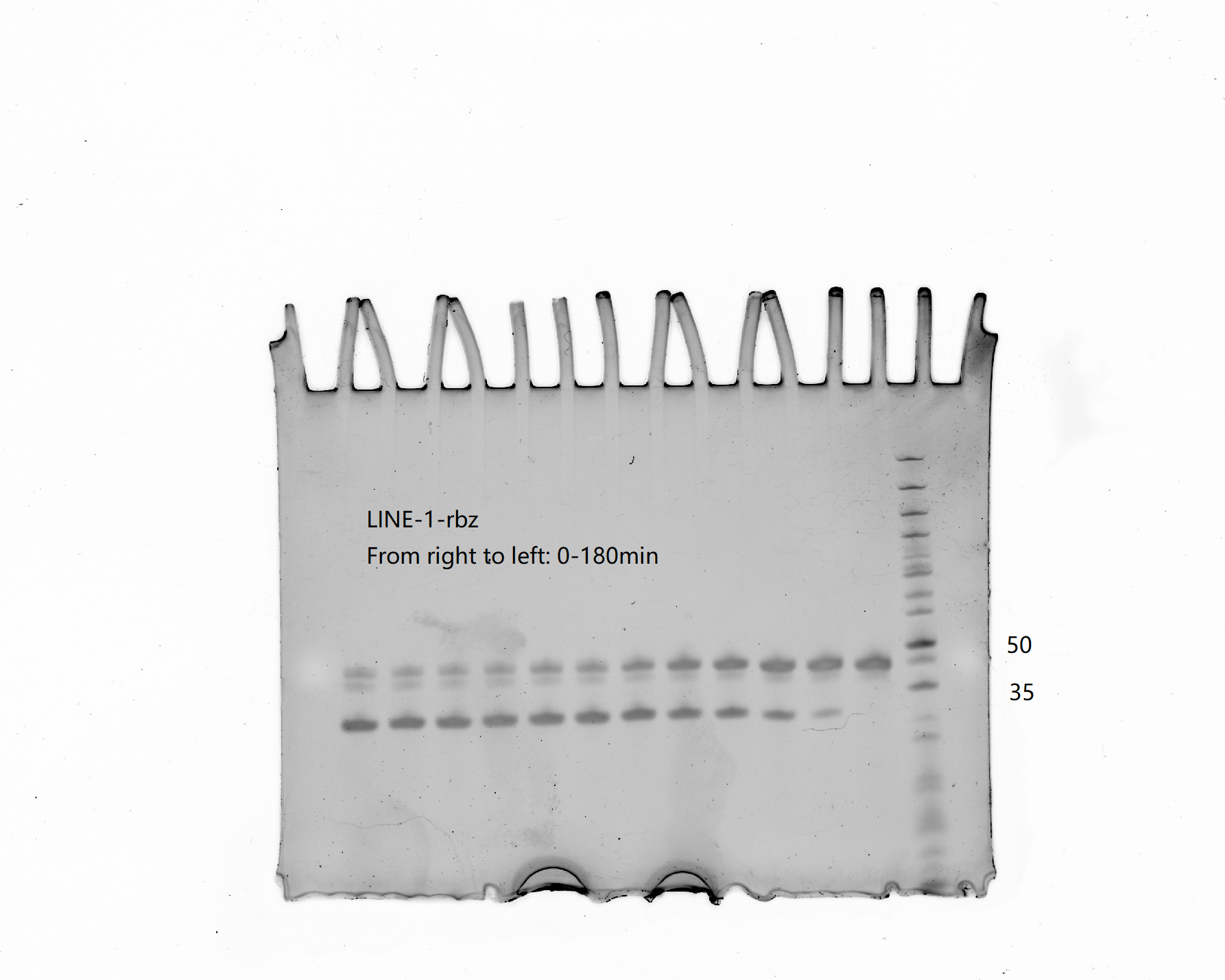

Supplement: Figure 5—figure supplement 5—source data 2. [file elife-90254-fig5-figsupp5-data2.zip › Figure 5-figure supplement 5 labeled 1.tif]

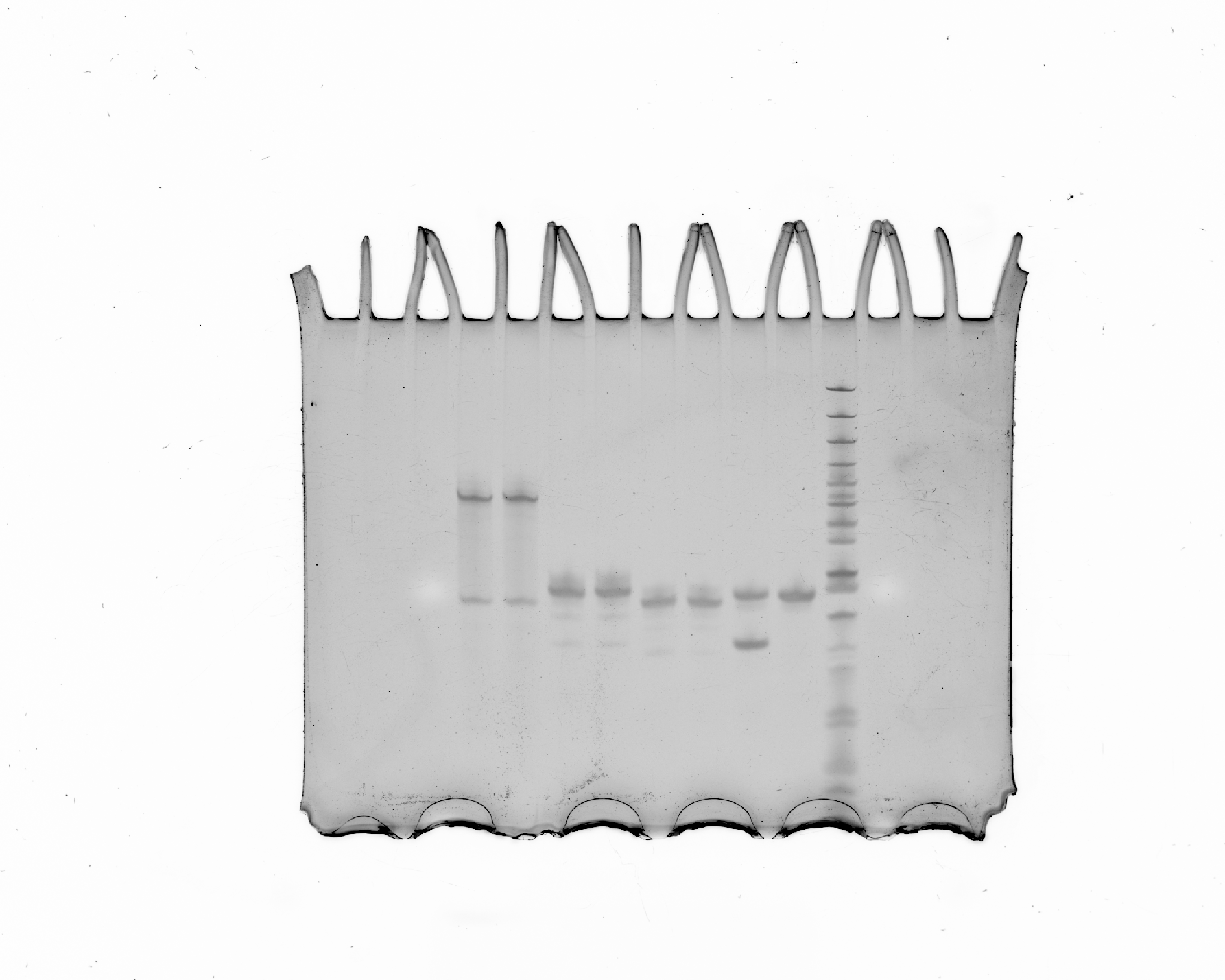

Supplement: Figure 6—source data 1. [file elife-90254-fig6-data1.zip › Figure 6 raw 2.tif]

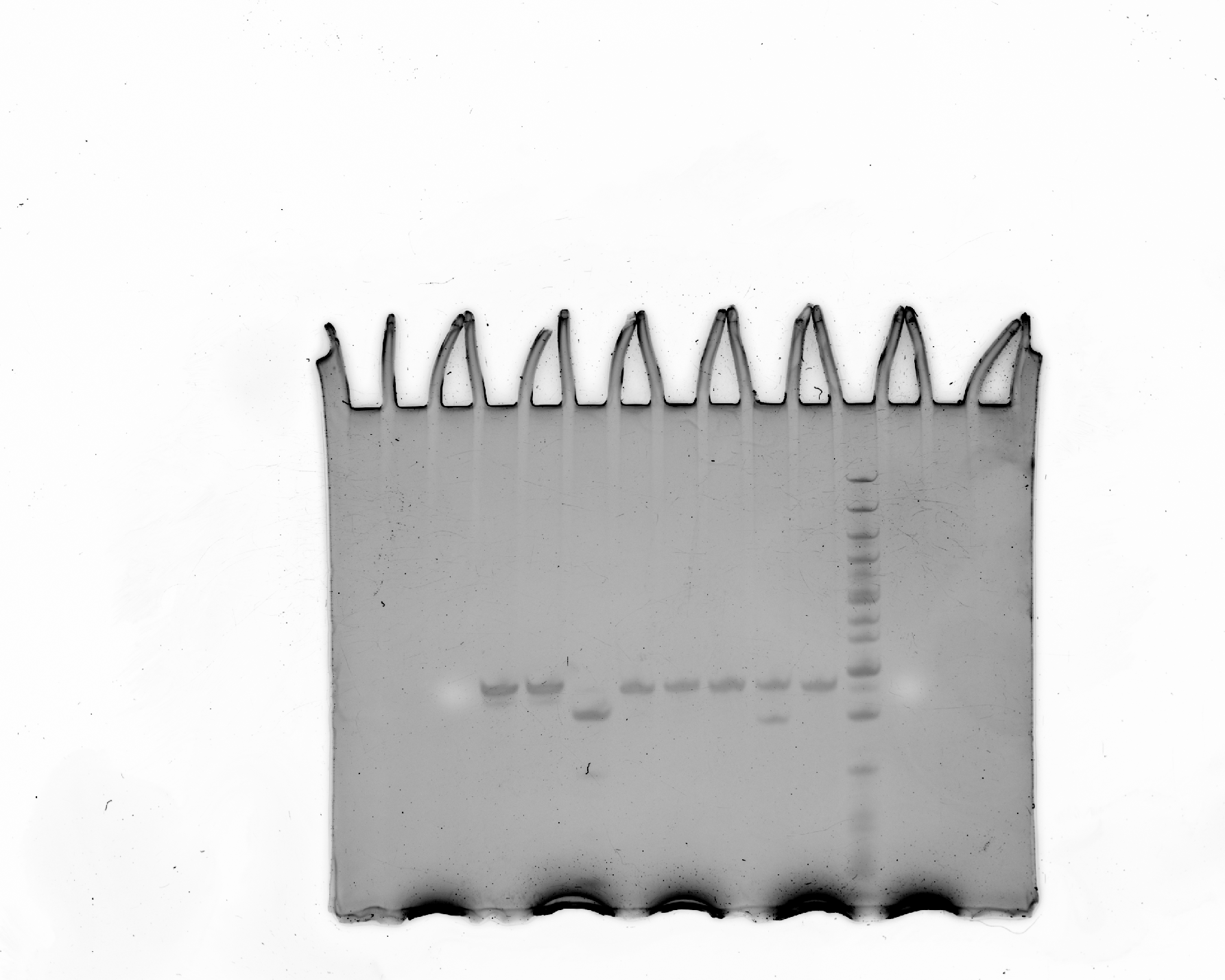

Supplement: Figure 6—source data 1. [file elife-90254-fig6-data1.zip › Figure 6 raw 1.tif]

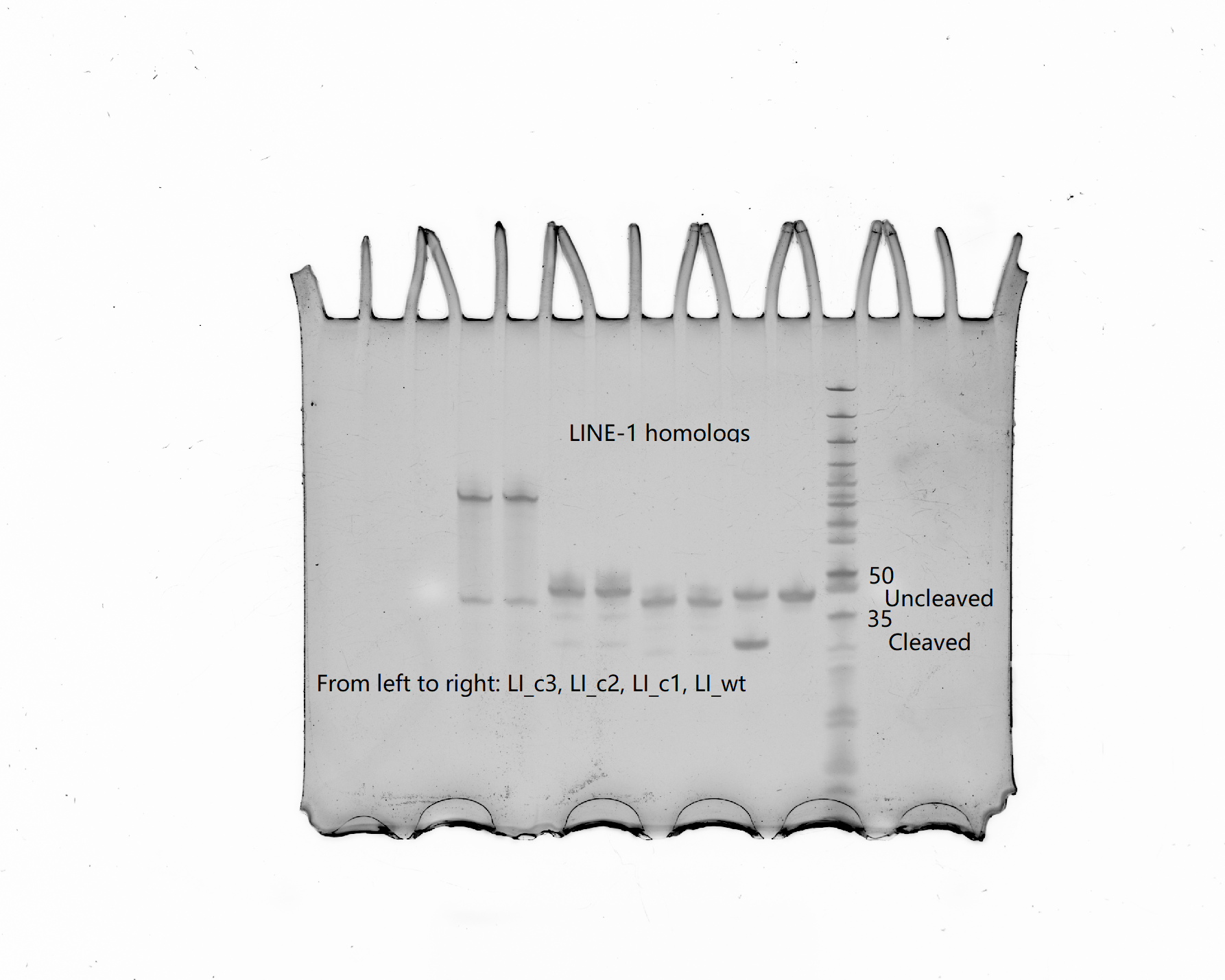

Supplement: Figure 6—source data 2. [file elife-90254-fig6-data2.zip › Figure 6 labeled 2.tif]

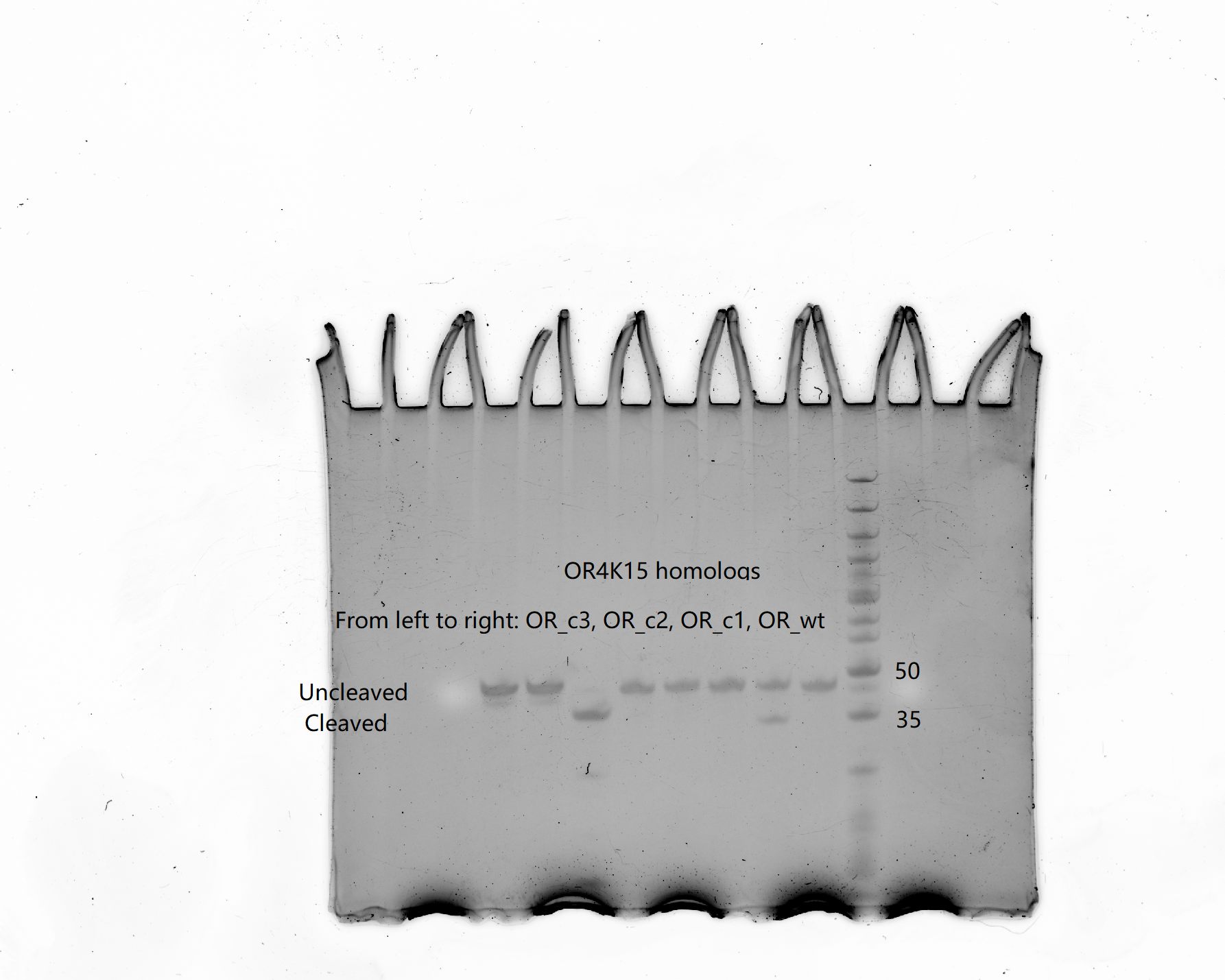

Supplement: Figure 6—source data 2. [file elife-90254-fig6-data2.zip › Figure 6 labeled 1.tif]
